# Diagnostic tools used in the evaluation of acute febrile illness in South India: a scoping review

**DOI:** 10.1186/s12879-019-4589-8

**Published:** 2019-11-13

**Authors:** Divyalakshmi Bhaskaran, Sarabjit Singh Chadha, Sanjay Sarin, Rajashree Sen, Sonia Arafah, Sabine Dittrich

**Affiliations:** 1 Foundation for Innovative New Diagnostics (FIND), Geneva, Switzerland; 2 London School of Hygiene and Tropical Medicine (LSHTM), London, UK; 3 Foundation for Innovative New Diagnostics (FIND), New Delhi, India; 4 Nuffield Department of Medicine, University of Oxford, Oxford, UK

**Keywords:** Fever, Acute febrile illness, Malaria, Scrub typhus, Dengue, Diagnostics, Tests, Diagnostic tests, Infectious diseases, Typhoid, Leptospirosis

## Abstract

**Background:**

Acute febrile illness (AFI) is characterized by malaise, myalgia and a raised temperature that is a nonspecific manifestation of infectious diseases in the tropics. The lack of appropriate diagnostics for the evaluation of AFI leads to increased morbidity and mortality in resource-limited settings, specifically low-income countries like India. The review aimed to identify the number, type and quality of diagnostics used for AFI evaluation during passive case detection at health care centres in South India.

**Methods:**

A scoping review of peer-reviewed English language original research articles published between 1946-July 2018 from four databases was undertaken to assess the type and number of diagnostics used in AFI evaluation in South India. Results were stratified according to types of pathogen-specific tests used in AFI management.

**Results:**

The review included a total of 40 studies, all conducted in tertiary care centres (80% in private settings). The studies demonstrated the use of 5–22 tests per patient for the evaluation of AFI. Among 25 studies evaluating possible causes of AFI, 96% tested for malaria followed by 80% for dengue, 72% for scrub typhus, 68% for typhoid and 60% for leptospirosis identifying these as commonly suspected causes of AFI. 54% studies diagnosed malaria with smear microscopy while others diagnosed dengue, scrub typhus, typhoid and leptospirosis using antibody or antigen detection assays. 39% studies used the Weil-Felix test (WFT) for scrub typhus diagnosis and 82% studies used the Widal test for diagnosing typhoid.

**Conclusions:**

The review demonstrated the use of five or more pathogen-specific tests in evaluating AFI as well as described the widespread use of suboptimal tests like the WFT and Widal in fever evaluation. It identified the need for the development of better-quality tests for aetiological diagnosis and improved standardised testing guidelines for AFI.

## Background

Fever in the tropics is a nebulous terminology. Due to the myriad clinical manifestations of fever, it is often broadly classified based on the duration of symptoms into: AFI and chronic fevers. As there is no consensus definition for the terms, chronic fever describes fevers lasting more than 14–21 days while AFI defines fevers lasting less than 21 days in duration as described in some publications [1]. AFI is synonymous with acute undifferentiated febrile Illness (AUFI), defined as: fevers resolving in 3 weeks lacking any localizable organ-specific signs or symptoms [2–4]. AFIs are often caused by infectious diseases in tropical, low-resource settings that have the highest burden of febrile illness [5–7].

Further, AFI can be classified based on aetiology as fever caused by malaria and non- malarial acute febrile illness (NMAFI) caused by other pathogens. The focus on malaria as a common cause of AFI in the developing world has led to the development of high-quality point-of-care testing (POCT) and rapid diagnostic tests (RDT) that aid in early diagnosis and timely therapeutic management of this illness. These developments have unmasked the under-recognized burden of NMAFI [1, 8–10].

AFI is a common cause of morbidity and mortality in children and adults in low and middle-income countries [11]. Aetiology of febrile illness in South Asia is reported to be caused principally by scrub typhus, dengue, malaria, typhoid and leptospirosis [2, 4, 6, 12–16]. India is a lower middle-income country (LMIC), with approximately 70% of its population living in rural areas [17]. Due to India’s geographical and seasonal heterogeneity, the lack of comprehensive surveillance, non-specific syndrome-based guidelines for fever management [18] and the lack of good-quality diagnostic tests, AFIs are poorly managed. In addition, due to the lax implementation of policies on prescription-based sales of antimicrobial agents, these are available cheaply leading to their extensive overuse, thus facilitating the development of antimicrobial resistance [19, 20]. In 2015, high-income countries (HIC) like the United States, France and Italy demonstrated a marginal increase in antibiotic consumption unlike the three leading middle-income countries - India, China and Pakistan that showed a drastic rise in antibiotic consumption. India surpassed China and Pakistan with an increase from 3.3 billion defined daily doses (DDD) of antibiotic consumption in 2000 to 6.5 billion DDD in 2015 (103%) compared to 79 and 65% increase in antibiotic consumption in China and Pakistan respectively [21]. 51–69% patients diagnosed with dengue in Chennai, who do not require antibiotics, were prescribed antimicrobial therapy-mostly cephalosporins and fluoroquinolones [12].

To gain a better understanding of the available and utilized tests in the Indian health system, this review intended to identify the diagnostic panel used for managing AFIs at health care centres of all levels in India. The objectives of this review were to identify the tests commonly used for the diagnostic evaluation and assessment of AFIs in patients attending for clinical care and to identify the number of diagnostic tests done per patient suffering from AFI. This is scoping review that aims to highlight the gaps in our understanding of AFI diagnosis and management from the available literature on AFI evaluation. The outcome of this study aims to emphasize diagnostic development needs as well as policy/guidelines and interventions that can support AFI diagnosis.

## Methods

A concept note was initially prepared outlining the key objectives, selection criteria and expected outcomes of the study. Based on this a search strategy was created and articles were screened for eligibility. The screening, assessment and data synthesis was done by DB. In case of challenging articles, it was discussed with SD to decide for article inclusion or exclusion. Both DB and SD were involved in the interpretation and analysis of data.

The key variables assessed in all studies were:

- Types of AFI investigated
- Type of diagnostic tests used for AFI evaluation
- The number of diagnostic tests used per patient in reaching a diagnosis of aetiology of AFI
- The setting of the studies: Public or private sector

### Selection criteria for publications

#### Case definition

For this review, any publication describing patients attending health care facilities with fever of acute onset (≤ 3 weeks duration), were included.

#### Study design

Peer-reviewed published literature describing diagnostic tests used for evaluation of AFI in patient care in South India which included: Cross-sectional studies, case-control studies, case reports and case-series.

#### Type of publications

Original research papers reporting AFI management in South India were included. Publications like reviews, mathematical models, articles on nosocomial infections/fevers, letters to the editor, short communications, conference abstracts and short notes were excluded.

#### Patient and setting characteristics

Patients of all age groups presenting with symptoms of AFI were included. As India is a large heterogeneous country, the review focused on the southern region of India that includes the states of Karnataka (KA), Andhra Pradesh (AP), Telangana (TS), Kerala (KE) and Tamil Nadu (TN). These included patients attending the Out-Patient Department (OPD) or emergency care department or patients admitted in tertiary care settings like inpatient departments (IPD) or intensive care units (ICU). Patients attending primary or community health centers based in rural, urban, public and private health care settings in South India were included.

#### Diagnostic tests in use

Diagnostic tests can be broadly classified into pathogen-specific and pathogen-nonspecific tests. Pathogen-specific tests provide an aetiological diagnosis of AFI. The types of pathogen-specific tests can be further categorized into antigen or antibody detection assays, molecular techniques of nucleic acid detection, and phenotypic tests for pathogen detection (e.g. smear microscopy for malaria, blood culture etc.)

Assessment of publications that documented the type, and number of pathogen-specific diagnostics used for evaluation, monitoring and prognosis of AFI were done.

#### Search strategy

Four electronic databases- Embase (1946), Medline (1946), PubMed (1996) and IndMED (1985) were searched for English language publications up to 13th July 2018. The databases were searched from their respective inception years to note the evolution in the types of diagnostic tools used for AFI diagnosis and to obtain maximum number of publications for analysis for this review. Three main search concepts were used: acute fever, diagnostic tests and India. Synonyms of fever like pyrexia, febrile illness were used. Individual causes of AFI were also inserted as search terms, e.g. scrub typhus, dengue etc. Similarly, synonyms for diagnostic tests like point of care testing, bedside testing etc. were used. Each term was searched as a keyword and MESH term. All the synonyms were connected by the Boolean operator OR. Truncation was used for terms like diagnosis and testing: diagnos* and test*. Each major concept was connected by the Boolean operator AND. Free text searching, manual hand-searching of journals and snowballing methods were used to obtain additional articles fitting the selection criteria of the review (Additional file 1: Table S1).

#### Data collection and extraction

The results of each search were exported to Endnote reference manager. Duplicates were removed. Full-text articles of potentially relevant studies were obtained, and studies included for the review were identified using the selection criteria. Data extraction was done using an adaptation of the Cochrane Effective Practice and Organization of Care (EPOC) data extraction form [22].

#### Quality assessment

This review appraised cross-sectional and case-control studies, case-series and case reports. Cross-sectional studies were appraised using the AXIS critical appraisal tool [23], while case-control studies, case-series and case reports were appraised using the Joanna Briggs Institute Reviewer’s manual (JBI tool) [24].

#### Data synthesis

A narrative synthesis was prepared using the data of the studies to describe type and number of diagnostics used in AFI evaluation. Descriptive and analytical statistics (Additional file 1: Supplementary data) of laboratory profiles of certain causes of AFI were reported. From the use of diagnostics, the commonly suspected pathogens of AFI were documented.

## Results

### Description of studies

**Fig. 1:**
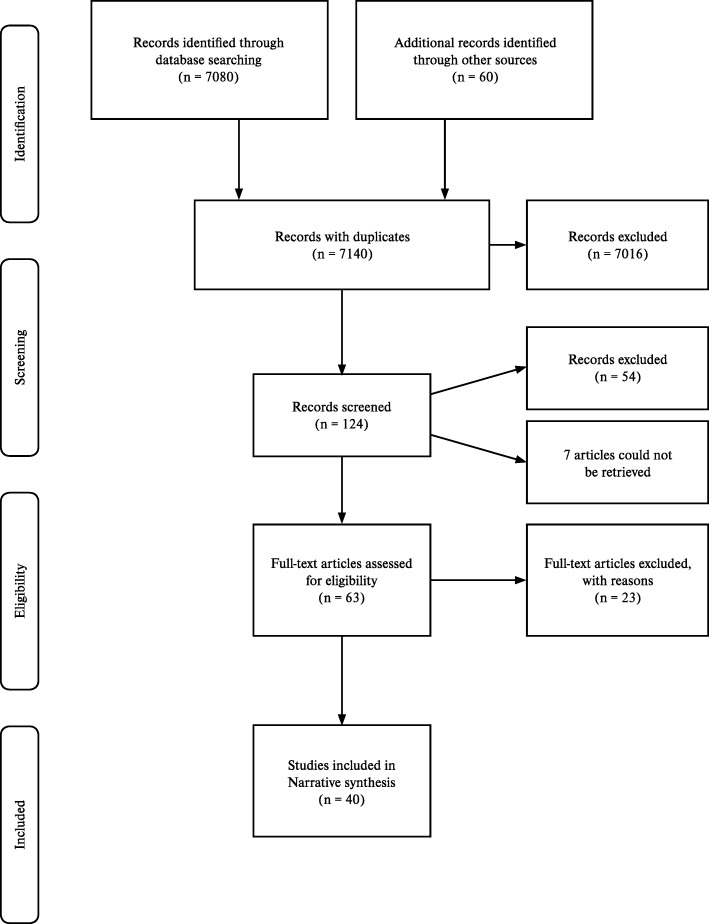
Adapted from PRISMA study flow diagram [source: Mohr et al. [47]]

In total, 7140 records with duplicates, were identified from four electronic databases and other methods. Based on title of the article, 7016 were excluded. Broadly, descriptive studies that documented the diagnostic approach to AFI and individual causes of AFI were included. Articles on comparative studies of different diagnostic tools for individual causes of AFI, or exclusively etiological studies of AFI, or AFI outbreak studies that only used a single test to confirm a pathogen were excluded. Etiological studies and outbreak studies that documented the diagnostic approach to AFI were included. 124 articles were analysed using abstracts from which 54 articles were excluded based on publication type (conference abstract, letter to the editor, short notes, short communications), setting (studies conducted in states other than KE, TN, KA, AP, TS). Seven studies were irretrievable despite sending emails to the authors or using library resources. From 63 articles considered for eligibility, 23 studies [3, 25–46] were excluded with reasons as summarised in ‘Characteristics of excluded studies’ (Additional file 1: Table S4). Totally, 40 studies were eligible for the review (Fig. 1).

### Study design, location and setting of studies

Of the 40 studies included for the review, 26 (65%) were cross-sectional studies [2, 16, 48–71], one (2.5%) was a case-control study [72], four (10%) were case series [73–76], and nine (22.5%) were case reports [77–85]. All studies were undertaken in tertiary care settings and were published between 2000 and 2018.

**Fig. 2:**
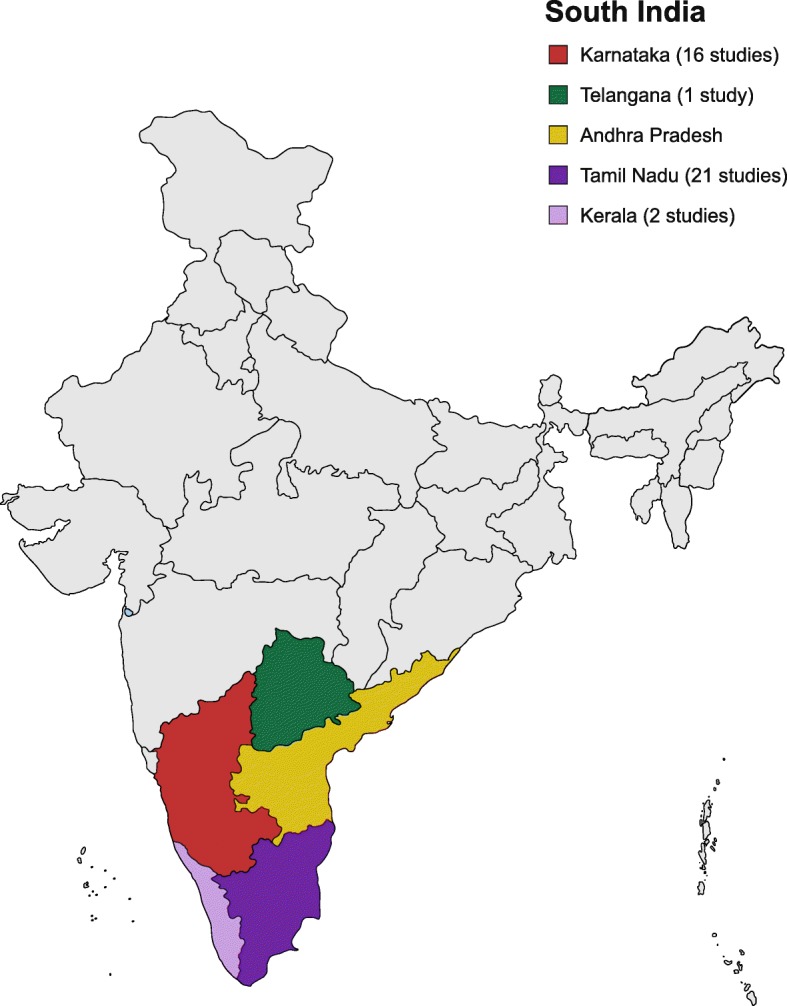
Distribution of studies in South India [Source: created from mapchart.net (https://mapchart.net/)]

Among the 26 cross-sectional studies, four studies were retrospective cross-sectional studies, one study was a retrospective and prospective cross-sectional study and the remaining 21 studies were prospective cross-sectional studies. The study Chrispal et al., 2010* was a sub-analysis of the larger study [16]. 21 studies were conducted in TN. Sixteen studies were conducted in KA. Two studies and one study were conducted in KE and TS respectively (Fig. 2, Additional file 1: Tables S5, S6, S7, S8, S9).

#### Patient populations

##### Case-series and case reports

Among four case-series (Table 1A), one study dealt with malaria in children [73], while two studies evaluated rickettsial infections in children [74, 75] and one study evaluated scrub typhus in adults [76].

**Table 1:** Characteristics of patient population described in case-series (A) and case reports (B)

A		
Name of study	Criteria for inclusion	Socio-demography
Kumar et al., 2008 [73]	4 cases: AFI diagnosed as malaria with radiological evidence of splenic involvement as a complication of malaria	Case-1: 42-year -old male
		Case-2: 38-year-old male
		Case-3: 65-year-old male
		Case-4: 15-year-old male
		All patients were from KA
Katoch et al., 2016 [74]	4 cases: AFI diagnosed as rickettsial infection with the presence of purpura fulminans	Case-1: 6-month-old infant female
		Case-2: 12-month-old female
		Case-3:7-month-old female
		Case-4:4 years old male
		All patients from KA
Saifudheen et al., 2012 [76]	2 cases: AFI with meningoencephalitis diagnosed as scrub typhus	Case-1: 45-year-old male, farmer
		Case-2: 30-year-old housewife
		Both patients from KE
Prasannan et al., 2017 [75]	4 cases: AFI with complication of gangrene diagnosed as a rickettsial infection	Case-1: 3-month-old female
		Case-2: 2-year-old male
		Case-3:12-month-old female
		Case-4: 8-year-old female
		All patients from KA
B		
Name of study	Case description and participant characteristics	
Manickam et al., 2014 [83]	A case of scrub typhus pneumonia in a 9-year-old female. Other causes of AFI ruled out before testing for scrub typhus. WFT > 1:160 antibody titre for OX-K antigen was positive while OX-2, OX-19 were negative	
Chandy et al., 2009 [78]	A case of hantavirus AFI in a 46-year-old male granary worker	
Devarajan et al., 2012 [79]	A case of a 55-year-old male with symptoms of AFI complicated by haematuria diagnosed as scrub typhus	
Thangaratham et al., 2006 [85]	A case of a 22-year-old male presenting with symptoms of AFI and diagnosed as coinfection of malaria with dengue	
Bhat et al., 2015 [77]	Clinical manifestations and lab parameters in a 22-year-old patient with AFI caused by 4 infections- dengue, *vivax* and *falciparum* malaria, Hepatitis A and E infection	
Jagdishkumar et al., 2016 [80]	A case of a 3-year-old male with AFI diagnosed with dengue and typhoid simultaneously	
Kakarapathi et al., 2014 [81]	Clinical manifestations of AFI caused by *vivax* malaria manifesting with neurological, haematological and renal complications in a 73-year-old woman	
Madi et al., 2014 [82]	Dengue-associated neurological manifestations in a 49-year-old male who presented with AFI	
Sitalakshmi et al., 2005 [84]	Description of AFI in a 27-year-old male diagnosed with *Plasmodium malariae*	

Among nine case reports (Table 1B), one evaluated scrub typhus [83] and another a coinfection of dengue and typhoid in a child [80]. Two studies evaluated mixed infections in adults [77, 85]. Of the remaining four studies, all of which were conducted in adult patients, one evaluated scrub typhus [79], one evaluated hantavirus infection [78], and the remaining two evaluated *Plasmodium vivax* malaria and dengue respectively [82, 84].

##### Cross-sectional and case-control studies

**Fig. 3:**
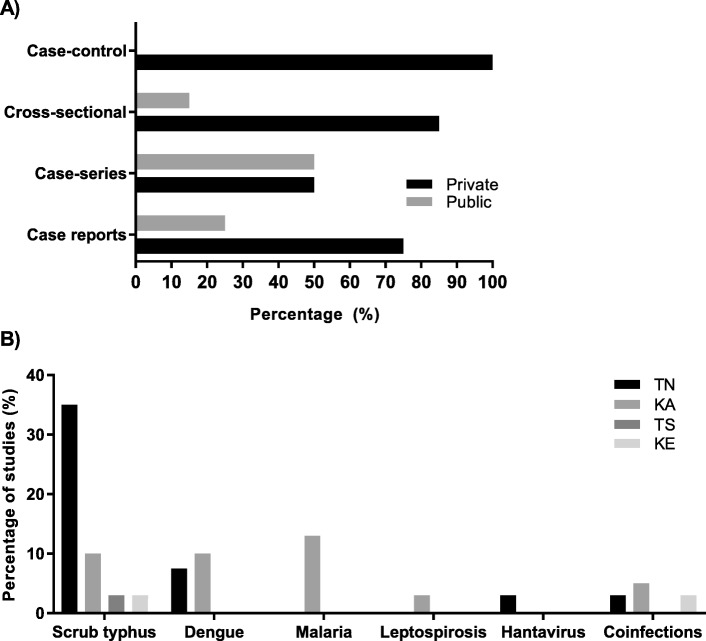
(**a**) Percentage distribution of different types of studies in private and public sector health care settings (**b**) Distribution of studies evaluating individual causes/pathogens of AFI among different states of South India

Three cross-sectional studies documented aetiology of AFI [2, 16, 55] and these were conducted in adults. Five studies conducted on dengue, were conducted in children [48, 56, 58, 62, 64]. One case-control study [72] was conducted in adults on the clinical and lab profile of dengue and scrub typhus coinfection. Two cross-sectional studies [54, 60] were conducted on malaria in adults while one was conducted on leptospirosis in adults [53]. Fifteen studies were conducted on scrub typhus. Four studies were conducted in children [50, 51, 57, 59]. The remaining studies were mainly conducted in adults (Additional file 1: Table S10) [49, 52, 61, 63, 65–71]. Across all studies, the setting was predominantly in the private sector with 85% cross-sectional studies and 75% case reports conducted in private settings (Fig. 3a). 35% of studies in TN evaluated scrub typhus while 13 and 10% in KA evaluated malaria and dengue respectively (Fig. 3b).

### Number of diagnostics used per patient in AFI evaluation in various studies

Out of 40 studies, 25 (63%) studies [2, 16, 48, 50, 51, 53, 55, 57, 65, 68, 70, 71, 73–85] used a combination of pathogen-specific and nonspecific tests to evaluate aetiology of AFI (Additional file 1: Table S11). The remaining studies evaluated specific types of AFI (e.g. scrub typhus, dengue).

Among these 25 studies, seven studies (28%) used > 10 pathogen-specific tests to ascertain the aetiology of AFI [16, 50, 65, 70, 76–78]. Four (16%) studies used < 5 specific tests [73, 74, 81, 84]. The remaining 14 (56%) studies used between 5 and 9 tests for etiological diagnosis of AFI [2, 48, 51, 53, 55, 57, 68, 71, 74, 79, 80, 82, 83, 85].

### Specific tests for aetiological diagnosis of AFI

Among 25 studies that tested for different causes of AFI, five diseases tested for commonly were: malaria (*n* = 24, 96%), dengue (*n* = 20, 80%), scrub typhus (*n* = 18, 76%), typhoid (*n* = 17, 68%), and leptospirosis (*n* = 15, 60%). Human immunodeficiency virus (HIV) testing was done in five (10%) studies for ascertaining cause of AFI [50, 55, 76, 77, 79], and in one study (4%), the Paul Bunnel test was used to rule out Epstein-Barr virus infection (EBV) [50]. Further, two studies (8%) tested for hantavirus as a cause of AFI [16, 78].

#### Malaria

**Fig. 4:**
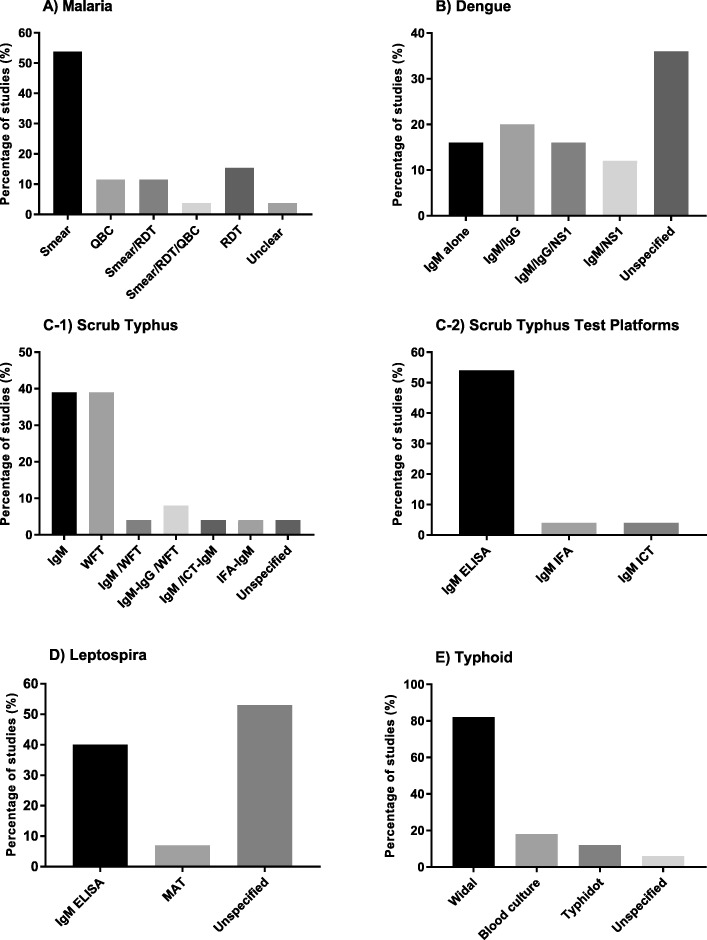
Types of tests used for diagnosis of specific causes of AFI: (**a**) Malaria (**b**) Dengue (**c1**) Scrub typhus (**c2**) Types of diagnostic platforms for IgM detection in Scrub Typhus (**d**) Leptospirosis (**e**) Typhoid

26 studies used malaria diagnostics [2, 16, 48, 50, 51, 53–55, 57, 60, 65, 68, 70, 71, 73, 74, 76–85] 14 studies (54%) used smear microscopy for parasite detection [2, 16, 48, 53, 54, 57, 73, 74, 76, 77, 79, 80, 84, 85]. and the remaining used RDT or quantitative buffy coat (QBC) alone or in combination for malaria diagnosis (Fig. 4a).

#### Dengue

**Fig. 5:**
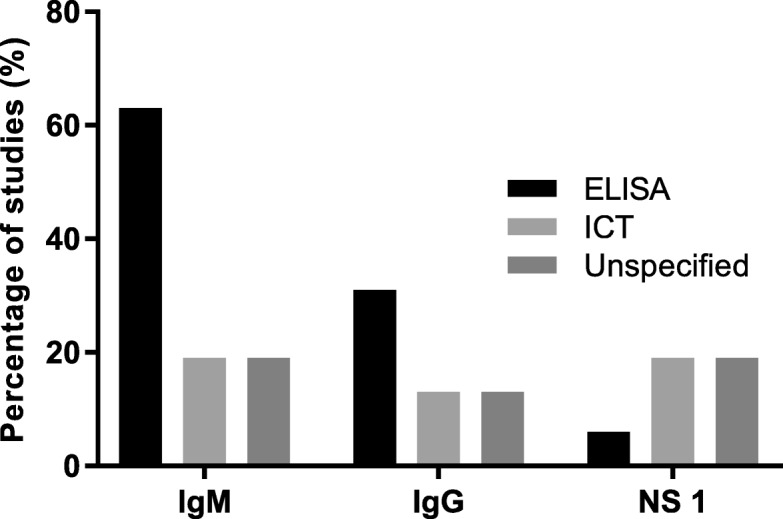
Percentage distribution of studies using different test platforms for IgM, IgG and NS 1 detection

Twenty five studies tested for dengue and of those, nine (36%) did not specify type of testing done for diagnosis while 16 (64%) studies documented the use of specific tests like dengue immunoglobulin M and/or G enzyme linked immunosorbent assay or immunochromatographic test (IgM, IgG, ELISA, ICT) and Non-structural protein 1 antigen (NS1) (ELISA or ICT) (Fig. 4b) [2, 16, 48, 50, 55, 56, 58, 62, 64, 70, 72, 77, 78, 80, 82, 85]. Among these 16 studies, ELISA was the commonly used test platform for IgM and IgG detection (Fig. 5).

**Fig. 6:**
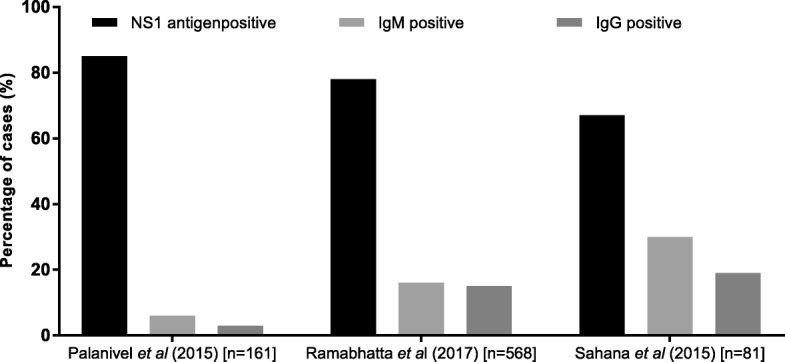
Distribution of dengue cases detected by NS1, IgM and IgG tests in 3 studies of different sample sizes (n)

In three studies [58, 62, 64], that had all test data available, the percentage of cases detected by NS1, IgM or IgG testing was reported (Fig. 6). The NS 1 test detected 67–85% of cases in these 3 studies. Ramabhatta et al., 2017 [62], a study conducted in a sample of 568 diagnosed cases of dengue, showed that IgG positive patients were more prone to complications than IgM positive patients. In this study, bleeding as a clinical manifestation showed statistical significance of *p* < 0.05 for patients with IgG antibodies.

#### Scrub typhus

**Fig. 7:**
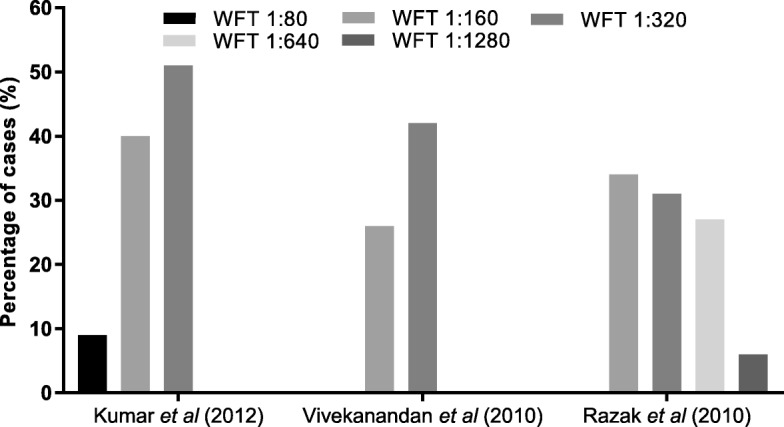
Percentage of scrub typhus cases diagnosed at different thresholds of antibody detection on WFT in 3 studies [50, 63, 71]

Among 40 studies, 26 (65%) studies tested for scrub typhus and other rickettsial infections. 25 (63%) out of these specified the types of aetiological diagnostics used [2, 49–52, 55, 57, 59, 61, 63, 65–72, 74–76, 78–80, 83]. The tests used included the WFT with OX-K antigen for diagnosis of scrub typhus (OX-2, OX-19 for other rickettsial infections), IgM or IgG detection by ELISA or ICT or immunofluorescence assay (IFA) (Figure 5C1, C2). Different WFT thresholds were described (Table 2) in various studies for a significant immunogenic response called the breakpoint titre (Fig. 7) [86, 87].

**Table 2:** Breakpoint titre threshold for positive WFT

Study	Positive threshold	Threshold for convalescent sera
Kumar et al., 2012 [50]	≥ 1:80	–
Mathai et al., 2003 [52]	≥ 1:80	–
Razak et al., 2010 [63]	≥ 1:160	4-fold rise starting from 1:40
Stephen et al., 2015 [65]	≥ 1:320	4-fold rise starting from 1:40
Subbalaxmi et al., 2014 [66]	≥ 1:80	–
Viswanathan et al., 2013 [70]	≥ 1:20	–
Vivekanandan et al., 2010 [71]	≥ 1:80	–
Katoch et al., 2016 [74]	≥ 1:320	4-fold increase in paired sera
Manickam et al., 2014 [83]	≥ 1:160	–
Prasannan et al., 2017 [75]	≥ 1:320	–

#### Leptospirosis

Fifteen out of 40 studies tested for leptospirosis. Among 15 studies, six (40%) studies used IgM ELISA in the diagnosis of leptospirosis (Fig. 4d) [2, 16, 55, 70, 78, 85] while one used microscopic agglutination test (MAT) [53].

#### Typhoid

17 out of 40 studies (43%) tested for typhoid. Among 17, three used blood culture [2, 16, 80], 14 used Widal [2, 48, 50, 51, 53, 57, 65, 70, 71, 74, 76, 79, 80, 83], while two used the Typhidot test to diagnose typhoid as a cause of AFI (Fig. 4e) [16, 78].

#### Aetiology of AFI

Three studies evaluated the causes of AFI [2, 16, 55]. The study Abhilash et al., 2016 [2] identified scrub typhus (35.9%), followed by dengue (30.6%) and malaria (10.4%) as the commonest causes of AFI. 17.5% cases of AFI had no identifiable cause despite all investigations. Similarly, Chrispal et al., 2010 identified scrub typhus (47.5%) followed by malaria (17.1%), enteric fever (8%) and dengue (7%) as commonest causes of AFI. 8% of AFI cases had no identifiable pathogen in this study. Finally, Muthaiah 2016, a study conducted in the ICU identified Dengue (18%), Leptospirosis (13%) as the main causes of AFI leaving 15% cases of AFI with unidentifiable pathogens.

### Quality of evidence

The risk of bias and quality of evidence is based on the appraisal of the studies using critical appraisal tools (Additional file 1: Tables S12-S20).

Among Four case-series, two studies (50%) [74, 76], showed a low risk of bias, the remaining two studies (50%) [73, 75], showed a moderate risk of bias. Among the nine case reports, seven (77.8%) [77, 78, 80, 82–85], showed a low risk of bias. Of the remaining two studies, one case report (11.1%) [79], showed a moderate risk of bias and the remaining one (11.1%) [81], showed a high risk of bias respectively. One case-control study had a high risk of bias [72]. Eight (30.8%) [2, 16, 49, 51, 55, 64, 69, 70], nine (34.6%) [53, 54, 56, 57, 59, 62, 65, 67, 68], five (19.2%) [48, 60, 61, 66, 71] and four (15.4%) [50, 52, 58, 63] studies out of 26 cross-sectional studies showed very low, low, moderate and high risk of bias, respectively (Additional file 1: Table S15).

## Discussion

The pattern of testing for AFI indicates that malaria is the most commonly suspected cause followed by dengue, scrub typhus, typhoid and leptospirosis. This is in line with a multicentre aetiological study of AFI in India where in a cohort of 1564 patients, malaria was the commonest cause of AFI followed by dengue, scrub typhus, bacteraemia and leptospirosis in decreasing order of frequency [35].

Based on the current data from southern India, malaria diagnosis by smear microscopy seemed more popular than antigen detection tests. As per the national guidelines for malaria diagnosis and management [88, 89], microscopy remains the gold standard for confirming malaria, though RDT are also mentioned as a method for rapid detection of the parasite. In the diagnosis of dengue, only a subgroup of studies used a combination of NS1 and IgM detection. The guidelines for dengue diagnosis and management prepared by the World Health Organization (WHO) [90], and at the national level by the Indian Council of Medical research (ICMR) (2015) [91], recommend the use of Polymerase chain reaction (PCR) for early detection of dengue. The IgM, IgG and NS 1 are other tests recommended in the diagnosis of dengue. There is no recommendation on the benefits of combined use of these diagnostics, however, there is strong evidence demonstrating high diagnostic accuracy with the combined use of NS1 antigen and IgM detection in dengue [92]. The sensitivity and specificity of NS1 antigen test is 49–59% and 93–99% respectively while that of IgM antibody test is 71–80% and 46–90% respectively and the median number of days of fever prior to admission sample collection was 5 days (interquartile range, 3 to 7 days) for both the above-mentioned tests [92]. The diagnostic accuracy for detection of IgM increases for late compared to early acute infection [93, 94]. The NS1 antigen is an early marker of acute infection and its combined use with IgM detection can provide a definitive diagnosis of 96.9–100% for samples obtained after 3 days of illness [95].

The ICMR guidelines (2015) [96], define a probable case of scrub typhus or rickettsial infection as a suspected clinical case showing a combination of a WFT titres of 1:80 or above to OX2, OXK and OX19, and a positive IgM ELISA test. Further, the guideline defines a confirmed case as being diagnosed either by PCR or IFA. In a review of scrub typhus diagnostics [97] pooled sensitivity/specificity of IgM detection by ELISA was 66%/92%. In addition, the IgM ELISA provides a diagnosis of scrub typhus as early as within 3–4 days of illness [98]. The WFT shows variable diagnostic accuracy as it detects IgM antibodies due to cross-reactivity between the antigens of *Proteus* and rickettsia and thus, the test can be positive when there is no infection. To counteract this problem, convalescent titres are often taken for an accurate diagnosis, however, this is challenging and makes acute diagnosis impossible. The breakpoint titre, which is the antibody concentration required for a significant immunogenic response [87], varies and is dependent on the prevalence of the disease. In one Thai study, the overall sensitivities and specificities of WFT at cut-off titres of 1:1280, 1:640, 1:160, 1:80 were 5.1%/100, 17.9%/100% and 52.1/93.3 and 79.5%/74.7% respectively [99]. The sensitivity and specificity of WFT differs with prevalence of disease which necessitates sero-surveys of antibodies to rickettsia in the population to inform diagnostic interpretation [98, 100, 101]. Compounding the diagnostic challenges, the WFT is generally positive in the second week of illness [101] and is, therefore, suboptimal for detection of early acute infection. Given all these limitations, it is concerning that 39% of studies used WFT in diagnosing acute scrub typhus.

Typhoid is a major cause of fever in Asia [102]. This work shows that published studies from southern India used mainly Widal test for diagnosis. Unfortunately, Widal is an unreliable diagnostic test due to its inherent variability, difficulty in establishing baseline titres for the population, cross-reactivity with other antigens and lack of reproducibility [103]. The sensitivity/specificity of Widal in a study conducted in Delhi was 57/83% [104] while another done in paediatric patients in Mysore was 34.1%/42.8% [105]. The Typhidot test has an average sensitivity of 84% and specificity of 79% as stated in a recent Cochrane review on typhoid RDT [106].

Across most studies, the use of WFT and Widal demonstrates the use of suboptimal pathogen-specific tests in the diagnosis of AFI. Similarly, smear microscopy for malaria lacks sensitivity at lower parasite densities [107]. Being labour-intensive, it may not be the ideal diagnostic tool in rural areas. Coinfections pose a challenge to physicians and can lead to falsely localising signs, e.g. jaundice which mimics infective hepatitis. This necessitates testing for additional pathogens as noted in two studies in this review [77, 85].

Although this review provides a unique view of the diagnostic workup for AFI in the Indian setting, it has significant limitations. As only one author has assessed the studies, a bias in study assessment could have been introduced. Similarly, peer-reviewed English language research articles were assessed, leading to a publication and language bias in this study. As all studies were conducted in tertiary settings, it is possible that patients with complications of AFI were evaluated due to a delayed diagnosis at peripheral health centres that led to patient referrals to tertiary centres. This may have contributed to numerous diagnostics being used due to the added difficulty of diagnosing AFI with complications. As the review assessed studies from South India, this study cannot be extrapolated to other parts of India. This is due to the spatial and temporal heterogeneity of the epidemiology of AFI and the diversity of health care practices in the Indian healthcare system. In addition, all the studies were conducted in teaching hospitals/ research settings which strive to follow best practice which may not be reflective of practices in other health care settings.

## Conclusions

Despite its limitations, the review provides a novel and comprehensive insight into the diagnostic evaluation of febrile illness in South India. The data show that a multitude of tests are being used, and it raises concerns regarding the potential patient management implications resulting from the use of suboptimal diagnostic tools. Similarly, the timely diagnosis of AFI is crucial in preventing mortality and morbidity. Thus, health care providers need guidance about the appropriate use of various tests and their utility at different stages of disease (e.g. IgM versus NS1 for diagnosing dengue). National or sub-national guidelines provided by health authorities can additionally support a more unified approach to fever diagnostics. It also highlights the need for further work to develop definitions for AFI and chronic fevers which might outline better algorithmic management with appropriate use of diagnostics in evaluating febrile illnesses. As a result of these improved testing algorithms patients will be treated with appropriate antibiotics in a timelier manner and unnecessary antibiotic use can be curbed.

## Supplementary information

**Additional file 1.** Complete search strategies employed for the review to screen and include articles. Descriptive and analytical statistics used in various papers. Table compilation of the types and number of diagnostic tests used for AFI evaluation. Quality assessment of the papers with risk of bias analysis using quality assessment tools. Characteristics of included and excluded studies.

## Data Availability

An additional data file containing supplementary data has been uploaded with the manuscript.

## Abbreviations

AFI: Acute febrile illness
AP: Andhra Pradesh
AUFI: Acute undifferentiated febrile illness
DDD: Defined daily doses
EBV: Epstein-Barr virus
ELISA: Enzyme linked immunosorbent assay
EPOC: Effective Practice and Organization of Care
HIC: High income country
HIV: Human immunodeficiency virus
ICMR: Indian Council of Medical Research
ICT: Immunochromatographic test
ICU: Intensive care unit
IgM/IgG: Immunoglobulin M/G
IPD: Inpatient department
JBI: Joanna Briggs Institute reviewer’s manual
KA: Karnataka
KE: Kerala
LMIC: Lower middle-income country
MAT: Microscopic agglutination test
NMAFI: Non-malarial acute febrile illness
NSP 1: Non-structural protein 1
OPD: Out-patient department
PCR: Polymerase chain reaction
POCT: Point-of-care test/testing
QBC: Quantitative buffy coat
RDT: Rapid diagnostic test
TN: Tamil Nadu
TS: Telangana State
WFT: Weil-Felix test
WHO: World Health Organization
