# Supplementary material for: Diagnostic tools used in the evaluation of acute febrile illness in South India: a scoping review
Source: BMC Infect Dis. 2019 Nov 13;19:970. doi: 10.1186/s12879-019-4589-8 (PMC6854686; doi:10.1186/s12879-019-4589-8)
Supplement: Supplementary file 1 — Additional file 1. Complete search strategies employed for the review to screen and include articles. Descriptive and analytical statistics used in various papers. Table compilation of the types and number of diagnostic tests used for AFI evaluation. Quality assessment of the papers with risk of bias analysis using quality assessment tools. Characteristics of included and excluded studies. [file 12879_2019_4589_MOESM1_ESM.docx]

# **Additional data file**

# **Supplementary data**

This file contains additional information that helps us explain the interpretations and analysis of our findings. It explains in detail the methodology in terms of the search strategies and subsequently the appraisal of articles considered for inclusion in the scoping review. It also contains the results of all papers collated together to understand the type and number of diagnostic tests used for fever evaluation.

## **Search strategy**

**Table 1. Embase® 1946-July 2018 and Ovid Medline® 1946-July 2018**

| S. No. | Search terms | Embase® 1946-July 2018 (Results) | Ovid Medline® 1946-July 2018(Results) |
| --- | --- | --- | --- |
| 1. | Scrub Typhus/ or Leptospirosis/ or Fever/ or Mucocutaneous Lymph Node Syndrome/ or Dengue/ or Dengue Virus/ or acute febrile illness.mp. or Chikungunya Fever/ | 264945 | 64535 |
| 2. | Rickettsia Infections/ or Dengue/ or Fever/ or acute fever.mp. or Chikungunya virus/ or "Fever of Unknown Origin"/ or Malaria/ | 315734 | 96375 |
| 3. | 1 or 2 | 339710 | 113464 |
| 4. | limit 3 to English language | 297515 | 90969 |
| 5. | Emergency Service, Hospital/ or Diagnostic Tests, Routine/ or HIV Infections/ or Influenza, Human/ or diagno* test*.mp. or Malaria/ or Mass Screening/ | 371192 | 449601 |
| 6. | (Point-of-care test* or POC test* or point of care test* or bedside test* or extra laboratory test* or near patient test*).mp. [mp=title, abstract, heading word, drug trade name, original title, device manufacturer, drug manufacturer, device trade name, keyword, floating subheading word, candidate term word] | 14596 | 4732 |
| 7. | Rural Population/ or Urban Population/ or INDIA/ or India*.mp. | 349573 | 249163 |
| 8. | limit 7 to English language | 329846 | 228777 |
| 9. | 5 or 6 | 383383 | 453312 |
| 10. | limit 9 to English language | 333000 | 401764 |
| 11. | 4 and 8 and 10 | 4017 | 2311 |
| 12. | limit 11 to year="1946 - 2018" | 4003 | 2308 |

**Table 2. PubMed 1996-July 2018**

| S. No | Search terms |
| --- | --- |
| 1. | Test*(text word) or test*(MeSH terms) |
| 2. | Diagnos*(text word) or diagnos*(MeSH terms) |
| 3. | Point of care (MeSH terms) |
| 4. | Fever (text word) or fever (MeSH major topic) |
| 5. | Febrile illness (text word) |
| 6. | Pyrexia (text word) or pyrexia (MeSH terms) |
| 7. | India (text word) or India (MeSH terms) |
| 8. | 1 and 2 and 3 and (4 or 5 or 6) and 7 |
| 9. | 8 restricted to English language |
| 10. | Limit 9 to 1996-July 2018 |
| Results: 10 | 523 |

**Table 3. IndMED 1985-July 2018**

| S. No. | Search terms (advanced) |
| --- | --- |
| 1. | Fever |
| 2. | Diagnosis |
| 3. | India |
| 4 | 1 and 2 and 3 |
| Results:4 | 246 |

**Table 4. Characteristics of excluded studies**

| **Name of study** | **Reason for exclusion** |
| --- | --- |
| Abrahamsen et al,2013 | The paper included patients coinfected with HIV and TB, HIV and cryptococcal infection, HBV with other infections. The paper included fevers of 21 days or more with the median duration of fever in the study of 5.4 weeks |
| Chandy et al,2009 | Does not document the diagnostic approach to AFI. |
| Chockalingam et al,2003 | Does not document diagnostic approach to AFI. It is a study specifically documenting profile of Rheumatic fever |
| Gopakumar et al,2018 | Documents hepatopathy caused by malaria and artesunate overdose, therefore it is not a documentation of the diagnostic approach to AFI |
| Ganesh et al,2013 | Does not document diagnostic approach or evaluation of AFI |
| Haanshus et al,2016 | Documents aetiology of fever and not diagnostic approach to AFI |
| Harris et al,2001 | Diagnostic approach to AFI not documented completely, sample size could not be clearly justified in the sample. A high risk of bias in the study as the reasons why all the patients were not investigated in the same way is unexplained |
| Ittyachen et al,2015 | Diagnostic approach not documented. The tests for aetiological diagnosis not mentioned, the author must infer or guess which specific tests were used for diagnosis |
| Kalal et al,2016 | The descriptive statistics cannot be replicated and there is inconsistency in the results which cannot be explained by statistics |
| Kamarasu et al,2007 | A sero-epidemiological study that documents prevalence of disease and does not document diagnostic workup of AFI |
| Khan et al, 1989 | Does not document diagnostic workup to AFI |
| Morch et al,2017 | Does not document diagnostic workup to AFI |
| Muthusthupathi et al,1995 | Specific test for documenting cause of AFI was done outside India |
| Nalini et al,2013 | Specific test for aetiological diagnosis of AFI done outside India |
| Narayanan et al,2003 | The findings of this paper are already documented in the study Narayanan et al,2002 |
| Pothapregada et al,2015 | The publication type is a short correspondence |
| Prabhu et al,2016 | A selection bias of only patients with fever and acute kidney injury (AKI). Patients with fever and no AKI were excluded. |
| Ravinder et al,2018 | Does not document how other causes of AFI were excluded when cases of dengue and leptospirosis coinfection were recruited. The diagnostic workup of patients in the study was not documented |
| Shetty et al,2012 | Does not document the tests used for the diagnosis and monitoring of malaria as a cause of AFI. It only documents the severity of thrombocytopenia in different types of malaria |
| Shivbalan et al,2010 | Fever with a localising cause- An abscess |
| Sukumar et al,1975 | Does not document diagnostic workup to AFI |
| Varghese et al,2013 | Documents differences in ST meningitis with other causes of meningitis, this does not answer the review question |
| Yellanthoor et al,2013 | Documents only malaria diagnosis and haemoglobin in all patients. Does not document a detailed diagnostic approach to malaria as a cause of AFI |

**Table 5. Distribution of studies in various districts in TN, KA, AP, KE**

| TN | |
| --- | --- |
| Vellore | 7 cross-sectional studies and one case report |
| Pondicherry | 6 cross-sectional studies and one case-control study |
| Chennai | 4 cross-sectional studies and one case report |
| KA | |
| Bangalore | 3 cross-sectional studies, one case series and one case report |
| Mangalore | One cross-sectional study, one case series and 2 case reports |
| Manipal | 2 cross-sectional studies and one case report |
| Mysore | 2 cross-sectional studies and one case report |
| Davangere | One case series study |
| AP: One cross-sectional study was conducted in Hyderabad | |
| KE | |
| Alappuzha | One case report |
| Calicut | One case series |

**Table 6.** **Study setting: Case series**

| Name of study | Location | Name of setting | Public /private |
| --- | --- | --- | --- |
| Kumar, G et al,2008 | KA | Kasturba Medical College, Mangalore | Private |
| Katoch et al,2016 | KA | Jagadguru Jayadeva Murugarajendra Medical College, Davangere | Private |
| Prasannan et al,2017 | KA | Bangalore Medical College, Bangalore | Public |
| Saifudheen et al,2012 | KE | Department of Neurology, Medical College, Calicut | Public |

**Table 7. Study setting: Case reports**

| Name of study | Location | Name of setting | Public/Private |
| --- | --- | --- | --- |
| Chandy et al,2009 | TN | Christian Medical College, Vellore | Private |
| Manickan et al,2014 | TN | Employees State Insurance-Postgraduate Institute of Medical Sciences and Research, Chennai | Public |
| Bhat et al,2015 | KA | Kasturba Medical College, Mangalore | Private |
| Jagdishumar et al,2016 | KA | Jagadguru Sri Shivarathreeshwara Medical College, Mysore | Private |
| Kakaraparthi et al,2014 | KA | Kasturba Medical College, Manipal | Private |
| Madi et al,2014 | KA | Kasturba Medical College, Mangalore | Private |
| Sitalakshmi et al,2005 | KA | Saint John’s Medical College, Bangalore | Private |
| Thangaratham et al,2006 | KE | T.D. Medical College,Alappuzha | Public |

**Table 8. Study setting and duration: Cross-sectional studies**

| Name of study | Location | Name of setting | Study period | Duration of Study(months) | Private/Public |
| --- | --- | --- | --- | --- | --- |
| Abhilash et al,2016 | TN | Christian Medical College, Vellore | October 2012- September 2013 | 12 | Private |
| Chacko et al,2008 | TN | Sri Ramachandra Medical College and Research Institute, Chennai | 1^st^ September 2005-31^st^ December 2005 | 4 | Private |
| Chrispal et al,2010 | TN | Christian Medical College, Vellore | January 2007- 2008 | 12 | Private |
| Kumar et al,2012 | TN | Jawaharlal Institute of Postgraduate and Medical Education and Research, Pondicherry | February 2011-2012 | 12 | Public |
| Premraj et al,2018 | TN | Chettinad Hospital and Research Institute, Kanchipuram, Chennai | June 2015- May 2016 | 12 | Private |
| Varghese et al,2006 | TN | Christian Medical College, Vellore | October 2002- February 2003 | 5 | Private |
| Varghese et al,2013 | TN | Christian Medical College, Vellore | August 2009-October 2010 | 15 | Private |
| Viswanathan et al,2014 | TN | Pondicherry Institute of Medical Sciences, Pondicherry | February 2011-January 2012 | 12 | Private |
| Varghese et al,2014 | TN | Christian Medical college, Vellore | 2005-2010 | 60 | Private |
| Chrispal et al,2010* | TN | Christian medical College, Vellore | January 2007- 2008 | 12 | Private |
| Narayanan et al,2002 | TN | Stanley Medical College, Chennai | 9^th^ October 2001-31^st^ December 2001 | 3 | Public |
| Palanivel,H et al,2015 | TN | Pondicherry Institute of Medical Sciences, Pondicherry | October 2012-December 2012 | 3 | Private |
| Vivekanandan et al,2010 | TN | Pondicherry Institute of Medical Sciences, Pondicherry | April 2006-2008 | 24 | Private |
| Stephen et al,2015 | TN | Mahatma Gandhi Medical College and Research Institute, Pondicherry | September 2012-March 2013 | 7 | Private |
| Mathai et al,2003 | TN | Christian Medical College, Vellore | October 2001-February 2002 | 5 | Private |
| Narayanaswamy et al,2016 | TN | Sri Manakula Vinayagar Medical College and Hospital, Pondicherry | November 2014-March 2015 | 5 | Private |
| Palanivel, S et al, 2012 | TN | Institute of Child Health and Hospital for Children, Chennai | October 2010-March 2011 | 6 | Public |
| Manjunath et al,2017 | KA | Jagadguru Sri Shivarathreeshwara Medical College, Mysore | December 2011-November 2012 | 12 | Private |
| Muthaiah et al,2016 | KA | Jagadguru Sri Shivarathreeshwara Medical College, Mysore | July 2013-December 2013 | 6 | Private |
| Poovathingal et al,2014 | KA | Kasturba Medical College, Manipal | May 2009-January 2011 | 21 | Private |
| Razak et al,2010 | KA | Kasturba Medical College, Manipal | January 2009-December 2009 | 12 | Private |
| Mathew et al,2006 | KA | National Institute of Mental Health and Neurosciences, Bangalore | October 98-2000: Retrospective  November 2000-2003: Prospective | 60 | Public |
| Ramabhatta et al,2017 | KA | Shifaa Hospital, Bangalore | July 2011- 2016 | 60 | Private |
| Sahana et al,2015 | KA | Sapthagiri Institute of Medical Sciences and Research Centre, Bangalore | July 2012-February 2013 | 8 | Private |
| Muddaiah et al,2006 | KA | Justice KS Hegde Charitable Hospital, Mangalore | 1^st^ September 2002-31^st^ August 2004 | 24 | Private |
| Subbalaxmi et al,2014 | AP | Nizam’s Institute of Medical Sciences, Hyderabad | August 2011-December 2012 | 14 | Public |

**Table 9. Study setting: Case-control studies**

| Name of study | Location | Name of setting | Study period | Duration of study (months) | Public/ Private |
| --- | --- | --- | --- | --- | --- |
| Basheer et al,2014 | TN | Pondicherry Institute of Medical Sciences, Pondicherry | January 2010-July 2014 | 54 | Private |

**Table 10. Characteristics of participants: cross-sectional and case-control studies**

| Name of study | Aim of study | Inclusion criteria | Exclusion criteria | Final Sample size  Mean (standard deviation-SD)/ Median (Interquartile range-IQR) Age(years)  Gender-Females(F) Males(M) | Sociodemographic  characteristics |
| --- | --- | --- | --- | --- | --- |
| Abhilash et al,2016 | Investigate causes of AFI, clinical predictors, and the seasonal trend through the year | Age >15 years with fever duration 3-14 days, with no focal sign of infection  Informed consent obtained  Patients attending medicine or emergency OPD | Fever due to non-infectious causes-haematological malignancy, Autoimmune diseases, patients on immunosuppressants | 1258  Mean Age (SD)=37.4(20) | Housewives: 31.7%  manual labourers/ Agricultural workers: 31%  Students: 17.2% |
| Chacko et al,2008 | Presentation of clinical, radiological and lab profile in suburban children<18 years from a tertiary centre with DF to determine predictive factors for Dengue Shock Syndrome (DSS) | Children aged>18 years with clinical features of Dengue confirmed with WHO criteria | Cases of fever diagnosed as Malaria, Leptospirosis, typhoid etc. | 73  Mean Age=7.87  M: F=1.52:1 | 16(21.92%) patients were infants. Youngest patient: 1 month old.44 males and 29 females in the sample |
| Chrispal et al, 2010 | Identify regional aetiology of AFI and describe disease-specific profiles that would help clinicians reach diagnoses | Adults aged> 16 years with fever >101ºF for 5-21 days with no focal evidence of infection. Patients requiring hospitalisation and providing informed consent | Patients suffering from HIV, haematological malignancies or Autoimmune diseases or on immunosuppressants | 398  Mean age (SD)=39.5(16.9)  M=242(60.1%), F=156(39.9%) | TN=66.3% AP=30.9% unemployed=33.4% labourers/farmers=38.7% |
| Kumar et al,2012 | Description of clinical profile and complications in children with Scrub typhus (ST) | Children aged<12 years with fever>5 days without identifiable focus of infection | Other causes of AFI | 35  Mean age=6.3 years  M=21(57%) F=15(43%) | Cuddalore=15 cases Villupuram=7cases Gingee=7 cases Rural areas of Pondicherry=2 cases |
| Premraj et al,2018 | Report the clinical profile, complications and risk factors associated with severe illness in patients with ST, outside the ICU | All age groups with AFI diagnosed as ST based on clinical features and Laboratory diagnosis-ST immunoglobulin M Enzyme linked immunosorbent assay (IgM ELISA) | Other causes of AFI | AFI patients=558  ST patients=50  Mean age (SD)= 39.6(20.5)  M=19(38%)  F=31(62%) | Rural areas=9(18%)  Semi urban=22(44%)  Urban=19(38%) |
| Varghese et al, 2006 | Derivation of clinical algorithm for diagnosing ST and determining predictors of bad prognosis among hospitalized patients with fever. | Patients aged >12 years with fever of 5-30 days duration hospitalized and diagnosed with ST using ST IgM ELISA | All other aetiologies of AFI ruled out | AFI=207  ST patients=50 (24.15%)  Median age (IQR)=36.5(12-75) | Farmers with low income=40(80%) |
| Varghese et al,2013 | Description of clinical and laboratory manifestations, genetic variability, and outcomes of ST | Patients aged>15 years hospitalized and diagnosed with ST by ST IgM ELISA | Other causes of AFI | ST patients=154  Mean age (SD)=46(15)  M=81(54%), F=73(46%) | Agricultural workers and housewives=77% |
| Viswanathan et al, 2013 | Comparison of characteristics of ST without meningitis with ST with meningitis | Adult cases aged > 16 years diagnosed with ST admitted in hospital  Confirmed cases of ST selected based on positive test on IgM ELISA, Weil-Felix test (WFT), presence of eschar or a combination of all three in a patient with AFI | Other causes of AFI | ST cases=65, 4 cases of other rickettsial infections  ST without meningitis, Mean age (ST without meningitis) (SD)=41.27(14.64)  Mean age (ST with meningitis) (SD)=41.82(17.67)  Number of patients-ST with meningitis: 17(26%)  M: F=10:7 | - |
| Varghese et al,2014 | Description of clinical aspects of ST for better clinical/lab profile of ST as a cause of AFI | Patients aged>16 years admitted with ST between 2005-2010  Diagnosis confirmed by ST IgM ELISA and/or presence of an eschar with PCR confirmation | Other causes of AFI | ST patients admitted=623  Mean age (SD)=45(15)  M: F=48%:52% | Agricultural labourers=41% |
| Chrispal et al,2010* | Delineation of clinical profile and predictors of mortality in patients with ST in South India | Same as Chrispal et al,2010 as it is a sub analysis of that study: Case definition for ST:  1.AFI and the presence of eschar and ST IgM ELISA positive or;  2.AFI and positive ST IgM ELISA with defervescence within 48hrs of initiation of Doxy or;  3. AFI with ST IgM ELISA seroconversion on convalescent sera or;  4. AFI and positive ST IgM ELISA with other serologies negative | Other causes of AFI | 398 patients of AFI of which 189(47.5%) diagnosed with ST included in study  Mean age (SD)=45.4(17.2)  M=100(52.9%)  F=89(41.9%) | Unemployed/housewife=42.9%  Unskilled labourer/farmer=38.8% |
| Narayanan et al,2002 | Document various manifestations and gather descriptive data of Dengue fever (DF) | Case definition: Probable cases of dengue by clinical suspicion, any AFI with one of the following: 1. myalgia 2. Headache 3. Retro-orbital pain 4. bleeding 5. altered sensorium 6. Shock 7. Low Platelet count  Children positive for dengue IgM ELISA alone or IgM-IgG  Informed consent taken | Other causes of AFI | 89 dengue suspects, 59 serologically confirmed  Mean age (SD)=6.76(3.19)  Age range=7months-12 years  Modal age group=5-6 years (14 children)  M=31(52.4%)  F=28(47.6%) | - |
| Palanivel et al, 2015 | Correlation of non-structural protein (NS1) and IgM detection with clinical spectrum of dengue infection among paediatric patients | All serologically confirmed cases of paediatric dengue aged<14 years admitted to tertiary care centre during the outbreak from October-December 2012 | Dengue suspects not serologically confirmed with dengue  All adults above 14 years of age with dengue  Children below 14 years of age with other causes of AFI | 429 dengue suspects  161(38%) serologically confirmed  Age group 5-13 years=68%,  1-5 years=29%,  Less than1 year=3%  M=52% F=48% | - |
| Vivekanan--dan et al,2010 | Describe diverse clinical and lab manifestations of ST | Patients with AFI aged > 12 years diagnosed with ST | Other causes of AFI | ST cases=50  Age range=14-19 years  M=22(44%), F=28(46%) | Most cases from rural areas of Pondicherry and nearby districts |
| Stephen et al,2015 | Analysis of clinical findings with presumptive ST, interpretation of hemogram, biochemical tests and ST specific serological tests such as rapid immunochroma-tographic test (RICT), ST IgM/IgG ELISA*,* WFT in paired serum samples | Patients with high grade fever with or without chills and rigour  Fever with rash/eschar/hepatosplenomegaly/jaundice/Lymphadenopath-y/thrombocytopenia;  Fever with constitutional symptoms like malaise, myalgia, nausea, vomiting, fever with capillary leak syndrome (pleural effusion, ascites, pedal oedema), fever with bleeding diathesis (petechia, purpura)/fever with shock  Patients who voluntarily provided acute and convalescent blood samples | Fevers due to HIV, lymphomas, malignancies and fevers with duration>4 weeks | ST suspects=45 Confirmed ST cases=28  Mean age (SD)=31.36(21.44)  youngest patient=1 year  oldest patient=89 years  M: F=1:1 | - |
| Mathai et al,2003 | Description of an outbreak of ST | AFI patients confirmed as ST with WFT | Other aetiologies of AFI | AFI=300, ST=28  Mean age (SD)=38.9(16-65)  M=15(55.56%), F=12(44,44%) | 60% belonging to low income groups  Patients from Vellore and neighbouring districts |
| Narayansw-amy et al,2016 | Evaluation of clinical and lab profile of paediatric ST in rural South India | Children aged between 6 months- 12 years admitted to paediatric ward with fever>5 days duration  ST IgM positive individuals included in the study | Other causes of AFI | AFI=448, ST=117  Mean age=6years, 6 months  Age range- 6months-12 years  M: F=1:1 | - |
| Palanivel et al,2012 | Evaluation of clinical profile and outcome of serologically confirmed ST cases admitted in an urban referral centre | Children<12 years admitted with serological confirmation of ST by ST IgM ELISA | Other causes of AFI | ST cases=67  46(68.65%) children in the age range of 1-6 years  8(11.94%) <1year age group  13(19.4%)> 6 years age  The patient with lowest age=60 days  In the age group=2months-11yrs  M: F=1:1.3 | Neighbouring districts around AP and rest of cases from TN=9(13.43%), Chennai=24(35.82%) Vellore=34(50.75%)  Children from urban area=39(58.2%)  Children from rural area=28(41.79%) |
| Manjunath et al,2017 | To ascertain incidence of ST using Immunofluorescence assay (IFA) in children with AFI | Children with fever >7 days duration admitted for AFI; serologically confirmed ST included in study | Other causes of AFI | AFI=857,  ST= 27  Age range= 1-15 years | - |
| Muthaiah et al,2016 | Study of aetiology and outcome of AFI patients developing multiple organ dysfunction syndrome (MODS) | Age>18 years, patient in a critical care setting with documented or history of fever Dysfunction and/or failure of 2 or more organs Organ dysfunction persisting for more than 24 hrs | Pregnancy, AFI associated with<2 organ dysfunction, chronic illness like DM, malignancies, IHD, Post-operative, post-traumatic or any surgical cases  Unable to give informed consent | AFI=213 cases  Cases fulfilling inclusion criteria=75  M=46(61.3%) F=29(38.7%) | - |
| Poovathing-al et al, 2014 | Document various clinical manifestations, lab parameters and outcomes of *falciparum* malarial infection | Patients aged>18yrs diagnosed with *Plasmodium falciparum* malaria | Other aetiologies of AFI | *falciparum* malaria cases=183  54% cases between 21-50 years of age  22.5% cases in the second decade of life  M=78%, F=22% | 49.2% cases from Udupi |
| Razak et al,2010 | Examine clinical and haematological profiles of patients with rickettsial fever and their outcomes | Patients with AFI diagnosed with rickettsial infections using WFT for OX-19, OX-2, OX-K. A titre of 1:160 or rise by 4-fold or more in titres on repeat testing starting 1:40 accepted as a positive result | Other aetiologies of AFI | ST cases=29  M=11(38%), F=18(62%) | - |
| Mathew et al,2006 | Evaluation of clinical features and lab profile of patients presenting with various neurological manifestations following leptospirosis in a tertiary care centre, to analyse outcome and prognostic indicators | leptospirosis diagnosed and with following criteria:  1.Patients with symptoms and signs referable to nervous system involvement  2.Biochemical evidence of hepatorenal dysfunction  3. Serological evidence of leptospiral infection by MAT | Patients with similar manifestations diagnosed to have cerebral malaria, enteric encephalopathy, viral encephalitis, TB meningitis or septicaemia | 31 patients confirmed on Microscopic agglutination test (MAT)  Mean age (SD)=36.4(14.3)  Age range=6-68 years  M=27 (87.1%), F=4 (12.9%) | Farmers=51.6%  manual labourers=22.6% |
| Ramabhatt-a et al,2017 | 1.Classify suspected dengue cases based on clinical features as per WHO guidelines with emphasis on serology  2. Identify early predictors of severe dengue | Children with fever 3-5 days duration with symptoms like dengue enrolled in the study with categorisation into 3 groups based on WHO 2012 criteria:  1.Dengue without warning signs(D)  2.Dengue with warning signs (DW)  3.Severe dengue (SD) | All other causes of AFI | Dengue cases=568  M=331(59%), F=237(41%) | - |
| Sahana et al,2015 | Description of clinical features and outcome of DF in children admitted during 2012 outbreak | Children presenting with symptoms of fever, myalgia, arthralgia, headache, vomiting, pain in abdomen, and bleeding manifestations with confirmed tests for dengue | Other causes of AFI | 81  M=55(67.9%), F=26(32.1%)  Commonest age group=5-15 years  Commonest age: Mean age=8 years | Majority of children were from urban areas=65.4% |
| Muddaiah et al,2006 | Description of demographic pattern and clinical presentation of malaria in adult patients admitted in hospital | Patients>15 years diagnosed smear positive on microscopy with symptoms of malaria. | Patients with fever testing negative for malaria but treated empirically for malaria and patients with clinical features mimicking malaria (malaria parasite negative) as in leptospirosis, dengue and sepsis, were excluded | 314  Outpatient data n=124  M=102(82%), F=22(18%)  Inpatient data n=190  M=153(80.5%),  F=37(19.5%) | OPD data  Mangalore district patients=106  Kerala and neighbouring districts=18  IPD data  Mangalore district patients=167  Kerala and neighbouring districts like Hassan, Chikmaglur, Bagalkot (North Karnataka) =23 |
| Subbalaxm-i et al,2014 | Describe Clinical features, laboratory profile, complications in patients diagnosed with ST | Patients aged>12 years with fever and confirmed ST  ST confirmed on WFT with titre of 1:80 and a positive ICT included in the study | All other causes of AFI | ST cases=176  Mean age (SD)=41(16)  M=105(59%)  F=71(40.3%) | Farmers=104(59.1%) Housewives=36(20.5%)  Skilled workers/students/businessmen=36(20.5%)  Most patients were from rural background working in open fields  Patients from rural part of AP= 170(96.6%) |
| Basheer et al,2016 | Description of clinical and laboratory features of Dengue and ST coinfection in a tertiary care hospital | Patients with fever with or without eschar, respiratory involvement in the form of pneumonitis and history of outdoor activity  Serological diagnosis of NS1 antigen for dengue confirmed and ST IgM ELISA diagnostic confirmation in these cases with co-infection  Cases with diagnostic confirmation of both infections were included | Other causes of AFI | 14 patients with coinfection  6 satisfied inclusion criteria  Mean age=42.5  M=67%, F=33% | - |

**Table 11. Diagnostics used in the evaluation of AFI**

| Name of study | Specific tests | Nonspecific tests | Total number of tests used |
| --- | --- | --- | --- |
| Abhilash et al,2016 | Blood culture (BC), ST, dengue, Leptospirosis IgM ELISA, widal, malaria smear | Liver function tests (LFT), Renal function tests (RFT), hemogram | 9 |
| Chacko et al,2008 | Dengue IgM ELISA, typhoid and leptospirosis serology, malaria smear, cerebrospinal fluid analysis (CSF) in patients with neurological manifestations, BC | LFT, RFT, Hemogram, Chest X-Ray (CXR), Platelet count (PC) | 11 |
| Chrispal et al,2010 | ST IgM ELISA, leptospirosis IgM ELISA, hantavirus IgM-IgG, typhidot IgM-IgG, dengue IgM-IgG ELISA, spotted-fever IgM ELISA, BC, malaria smear | Hemogram, LFT, RFT, CXR, PC | 16 |
| Kumar et al,2012 | Malaria smear, malaria histidine rich protein 2 (HRP2) RDT, widal, dengue IgM ELISA, leptospirosis serology, Paul Bunnel test, Urine culture (UC) and BC, tuberculin test, HIV ELISA, WFT OX-K titre of 1:80 threshold for diagnosis, CSF analysis in neurological manifestations | Hemogram, LFT, RFT, urine examination, echocardiogram (Echo), Electrocardiogram (ECG) and Creatine phosphokinase (CPK-MB) for cardiac complications | 19 |
| Premraj et al 2018 | ST IgM ELISA | Hemogram, RFT, LFT, PC, CXR | 6 |
| Varghese et al, 2006 | ST IgM and IgG ELISA, WFT OK-19, OX-K and OX-2 | Hemogram, PC, RFT, LFT | 6 |
| Varghese et al,2013 | Malaria quantitative buffy coat (QBC), dengue and leptospirosis serology, BC, ST IgM ELISA | Hemogram, RFT, LFT, CPK, CXR, abdominal ultrasonogram (USG) | 11 |
| Viswanathan et al,2013 | Leptospira serology (IgM), malaria antigen test, widal, dengue IgM-IgG, NS1 antigen, BC, ST IgM ELISA, WFT, CSF analysis | Hemogram, RFT, LFT, PC, CXR | 15 |
| Varghese et al,2014 | ST IgM ELISA, polymerase chain reaction (PCR) confirmation | Hemogram, RFT, LFT, PC, CXR, CPK | 8 |
| Chrispal et al,2010* | Dengue IgM-IgG ELISA, leptospira IgM ELISA, hantavirus IgM-IgG Qualitative assay, typhidot(IgM-IgG), spotted-fever IgM ELISA | Hemogram, PC, RFT, LFT, CXR, Arterial blood gas analysis (ABGA) | A sub-analysis of Chrispal et al,2010 study |
| Narayanan et al,2002 | Dengue IgM-IgG ELISA CSF in patients with neurological complications | Hemogram, PC, LFT, RFT, Urine examination, CXR | 7 |
| Palanivel,H et al, 2015 | NS1 antigen, IgM-IgG ELISA | Not documented except for PC | 3 |
| Vivekanandan et al,2010 | BC, widal, malaria RDT, leptospira and dengue serology, UC, CSF analysis, WFT | Hemogram, Urine examination, RFT, random blood sugar (RBS), LFT, CXR, Ultrasonogram (USG) abdomen | 15 |
| Stephen et al,2015 | BC, UC, widal, Leptospira and dengue serology, malaria smear, malaria antigen test, CSF analysis, ST ICT, ST IgM-IgG ELISA, WFT, convalescent sera at 14 and 21 days | Hemogram, LFT, RFT, PC, ABGA | 17 |
| Mathai et al,2003 | WFT using OX-K, OX-2, OX-19 antigen positive with cut-off threshold: 1:80 titre | Hemogram, PC, LFT, RFT, CXR | 6 |
| Narayanswamy et al,2016 | Widal, BC, UC, dengue serology, CSF analysis, ST IgM ELISA, malaria smear | Hemogram, LFT, RFT, CXR, Electrocardiogram (ECG), Echocardiogram (ECHO), USG Abdomen, ABGA, Creatine phosphokinase (CPK) | 16 |
| Palanivel,S et al,2012 | ST IgM ELISA | Hemogram, RFT, LFT, CXR | 4 |
| Manjunath et al,2017 | BC, Urine culture (UC), QBC malaria, dengue serology, widal, Mantoux test, IFA for ST, CSF analysis | Hemogram, LFT, RFT, CXR, USG abdomen and chest, CPKMB, ABGA, Echo | 17 |
| Muthaiah et al, 2016 | Dengue IgM ELISA, NS1 antigen, leptospira IgM, WFT, HIV ELISA, QBC malaria  Bacteriological assessment for relevant pathogens-body fluid assessment, grams stain, culture | Hemogram, Urine examination, RFT, LFT, ECG, ABGA, CXR, USG abdomen | 15 |
| Poovathingal et al, 2014 | Falcivax(malaria RDT), QBC, malaria smear | Hemogram, RFT, LFT, CXR, Coagulation profile, random blood sugar (RBS), Urine examination, ABGA | 12 |
| Razak et al,2010 | WFT, CSF | Hemogram, PC, LFT, RFT, CPKMB, CXR, USG abdomen, ECHO | 9 |
| Mathew et al,2006 | Peripheral blood smear (PBS) malaria, widal, BC, CSF analysis, MAT sera (30), CSF MAT (22) | RFT, LFT, Hemogram, PC, Computerised Tomography brain (CT) | 10 |
| Ramabhatta et al,2017 | NS1 antigen, IgM-IgG ELISA | Hemogram, LFT, RFT, Coagulation profile, USG abdomen, CXR | 9 |
| Sahana et al,2015 | Dengue NS1 antigen, IgM-IgG ELISA | Hemogram, Coagulation profile, LFT, RFT, RBS | 8 |
| Muddaiah et al,2006 | Malaria smear documented, tests for excluding other causes of AFI not documented | Hemogram, LFT, RFT | 5 |
| Subbalaxmi et al,2014 | WFT, ST ICT | Hemogram, PC, LFT, RFT, CXR | 7 |
| Basheer et al,2016 | Dengue NS1 antigen, dengue IgM ELISA, ST IgM ELISA | Hemogram, PC, RFT, LFT, Coagulation profile | 7 |
| Manickam et al, 2014 | Malaria antigen, dengue serology, widal, blood culture, WFT>1:160 titre for OXK, OX2, OX19, ST IgM ELISA | Hemogram, CRP, PC, CXR, USG | 11 |
| Chandy et al,2009 | Leptospira IgM, ST IgM ELISA, typhidot, dengue IgM ELISA, malaria antigen, BC, bone marrow aspiration, Blood samples (acute and convalescent) tested for IgM-IgG (hantavirus)  Polymerase chain reaction (PCR) of RNA extract from buffy coat sample targeting S genome of Hantavirus | Hemogram, coagulation profile, LFT, RFT, Urine examination, lactate dehydrogenase (LDH), CPK, CXR | 18 |
| Devarajan et al,2012 | BC, UC, Collagen disease workup, malaria smear, widal, leptospirosis, dengue and HIV serology, WFT | LFT, RFT, Urine examination, CXR, ECG, Echo, USG abdomen | 16 |
| Thangaratham et al, 2006 | Malaria smear, leptospira IgM, widal test, Anti hepatitis A and E titre, dengue IgM, BC | RFT, LFT, Hemogram, CXR, | 11 |
| Bhat et al,2015 | Dengue IgM ELISA, Hepatitis A, E (IgM kit), HIV, Leptospiral and, Rickettsia serology, Hepatitis B antigen (HBsAg), anti-Hepatitis C titre, BC, malaria smear | Hemogram, PC, LFT, CXR, ECG, USG | 16 |
| Jagdishkumar et al,2016 | Dengue IgM-IgG, malaria smear, WFT, BC, UC, widal | Hemogram, PC, LFT, RFT, Urine examination | 12 |
| Kakaraparthi et al,2014 | Malaria smear, card test for falciparum | Hemogram, PC, RFT, LFT | 6 |
| Madi et al,2014 | Dengue NS1 antigen, dengue IgM, CSF analysis, CSF anti- Japanese encephalitis (JEV) IgM antibodies, malaria test | Hemogram, PC, LFT, RFT, magnetic resonance imaging (MRI) brain, electroencephalogram (EEG), | 11 |
| Sitalakshmi et al,2005 | Malaria smear | Hemogram, PC, RFT, LFT | 5 |
| Kumar et al,2008 | Case-1: Malarial parasite fluorescent test  Case-2: Malaria smear  Case-3: Malaria smear  Case-4: Malaria smear | Case-1: Hemogram, RFT, urine examination, LFT, USG abdomen, CT scan  Case-2: Hemogram, Coagulation profile, Serum amylase, RFT, LFT, CT abdomen  Case-3: Hemogram, PC, LFT, RFT, MRI abdomen  Case-4: Hemogram, PC, LFT, RFT, USG abdomen | 6-8 |
| Katoch et al,2016 | All 4 cases tested for WFT OX-K, OX-2, OX-19, malaria smear, dengue serology, BC and widal | Case-1: Hemogram, LFT, RFT, Urine examination  Case-2: Hemogram, Coagulation profile, LFT, RFT, Urine examination  Case-3: Hemogram, LFT, RFT, urine examination  Case-4: Hemogram, CRP, RFT, LFT, Urine examination | 9-10 |
| Saifudheen et al,2012 | Both cases investigated for: WFT: OX-K, dengue, leptospirosis and HIV serology, widal, hepatitis A, B, E tests, CSF analysis, malaria smear | Case-1: Hemogram, PC, RFT, LFT, Urine examination, USG abdomen, CXR  Case-2: Hemogram, PC, RFT, LFT, Urine examination, USG abdomen, CXR, CT brain, EEG | 17-22 |
| Prasannan et al,2017 | WFT: OX-K done in all cases | All 4 cases: Hemogram, PC, CRP, RFT, LFT | 6 |

**Table 12.** **Quality assessment: AXIS tool for appraisal**

| S. No. | **Methods** | Yes/No/Unclear/Not applicable (NA) |
| --- | --- | --- |
| 1. | Were the aims /objectives of the study clear? |  |
| 2. | Was the study design appropriate for the aim? |  |
| 3. | Was the sample size justified? |  |
| 4. | Was the target/ reference population clearly defined? (is it clear who the research was about?) |  |
| 5. | Was the sample frame taken from an appropriate population base so that it closely represented the target/reference population under investigation |  |
| 6. | Was the selection process likely to select subjects/ participants that were representative of the target/reference population? |  |
| 7. | Were the measures undertaken to address and categorise non-responders? |  |
| 8. | Were the risk factors and outcome variables measured appropriate to the aims of the study? |  |
| 9. | Were the risk factors and outcome variables measured correctly using instruments/measurements that had been trialled, piloted or published previously? |  |
| 10. | Is it clear what was used to determine statistical significance and/or precision estimates? (e.g. p values, confidence intervals) |  |
| 11. | Were the methods (including statistical methods) sufficiently described to enable them to be repeated? |  |
|  | **Results** |  |
| 12. | Were the basic data adequately described? |  |
| 13. | Does the response rate raise concerns about non-response bias? |  |
| 14. | If appropriate, was information about non-responders described? |  |
| 15. | Were the results internally consistent? |  |
| 16. | Were the results presented for all the analyses described in the methods? |  |
|  | **Discussion** |  |
| 17. | Were the authors, discussions and conclusions justified in the results? |  |
| 18. | Were the limitations discussed? |  |
|  | **Others** |  |
| 19. | Were there any funding sources or conflicts of interest that may affect the authors' interpretation of the results? |  |
| 20. | Was ethical approval or consent of the participants attained? |  |

**Table 13.** **Appraisal of cross-sectional studies: METHODS**

| Study | Is the aim of the study clear? | Was the study design apt for the stated aim? | Was the sample size justified? | Was the reference population clearly defined? | Is the sample frame taken from an appropriate population base so that it closely represented the reference population under investigation? | Was the selection process likely to select subjects/ participants that were representative of the reference population? | Were the measures undertaken to address and categorise non-responders? | Were the risk factors and outcome variables measured appropriate to the aims of the study? | Were the risk factors and outcome variables measured correctly using instruments/measurements that had been trialled, piloted or published previously? | Is it clear what was used to determine statistical significance and/or precision estimates? | Were the method-s/st-atistical methods, sufficiently described to enable them to be repeated? |
| --- | --- | --- | --- | --- | --- | --- | --- | --- | --- | --- | --- |
| Abhilash et al,2016 | Yes | Yes | Yes | Yes | Yes | Yes | Yes | Yes | Yes | Yes | Yes |
| Chacko et al,2008 | Yes | Yes | Yes | Yes | Yes | Yes | unclear | Yes | Yes | Yes | Yes |
| Chrispal et al,2010 | Yes | Yes | Yes | Yes | Yes | Yes | NA | Yes | Yes | Yes | Yes |
| Kumar et al,2012 | Yes | Yes | No | Yes | Yes | Yes | NA | Yes | Yes | NA | Yes |
| Premraj et al,2018 | Yes | Yes | Yes | Yes | Yes | Yes | NA | Yes | Yes | Yes | Yes |
| Varghese et al,2006 | Yes | Yes | Yes | Yes | Yes | Yes | NA | Yes | Yes | Yes | Yes |
| Varghese et al,2013 | Yes | Yes | Yes | Yes | Yes | Yes | NA | Yes | Yes | Yes | Yes |
| Viswanathan et al,2013 | Yes | Yes | Yes | Yes | Yes | Yes | NA | Yes | Yes | Yes | Yes |
| Varghese et al,2014 | Yes | Yes | Yes | Yes | Yes | Yes | NA | Yes | Yes | Yes | Yes |
| Chrispal et al,2010* | Yes | Yes | Yes | Yes | Yes | Yes | Yes | Yes | Yes | Yes | Yes |
| Narayanan et al,2002 | Yes | Yes | Yes | Yes | Yes | Yes | NA | Yes | Yes | Yes | Yes |
| Palanivel H,et al,2015 | Yes | Yes | Yes | Yes | Yes | Yes | NA | Yes | Yes | Yes | No |
| Vivekanandan et al,2010 | Yes | Yes | Yes | Yes | Yes | Yes | NA | Yes | Yes | NA | Yes |
| Stephen et al,2015 | Yes | Yes | Yes | Yes | Yes | Yes | Yes | Yes | Yes | Yes | Yes |
| Mathai et al,2003 | Unclear | Yes | Yes | Yes | Yes | Yes | Yes | Unclear | Yes | No | Yes |
| Narayanaswamy et al,2016 | Yes | Yes | Yes | Yes | Yes | Yes | NA | Yes | Yes | NA | Yes |
| Palanivel S et al,2012 | Yes | Yes | Yes | Yes | Yes | Yes | NA | Yes | Yes | NA | Yes |
| Manjunath et al,2017 | Yes | Yes | Yes | Yes | Yes | Yes | NA | Yes | Yes | Yes | Yes |
| Muthaiah et al,2016 | Yes | Yes | Yes | Yes | Yes | Yes | Yes | Yes | Yes | Yes | Yes |
| Poovathingal et al,2014 | Yes | Yes | Yes | Yes | Yes | Yes | NA | Yes | Yes | Yes | Yes |
| Razak et al,2010 | Yes | Yes | Yes | Yes | Unclear | Yes | NA | Yes | Yes | NA | Unclear |
| Mathew et al,2006 | Yes | Yes | Yes | Yes | Yes | Yes | Yes | Yes | Yes | Yes | Yes |
| Ramabhatta et al,2017 | Yes | Yes | Yes | Yes | Yes | Yes | NA | Yes | Yes | Yes | Yes |
| Sahana et al,2015 | Yes | Yes | Yes | Yes | Yes | Yes | NA | Yes | Yes | Yes | Yes |
| Muddaiah et al,2006 | Yes | Yes | Yes | Yes | Yes | Yes | NA | Yes | Yes | NA | Yes |
| Subbalaxmi et al,2014 | Yes | Yes | Yes | Yes | Yes | Yes | NA | Yes | Yes | Unclear | Yes |

**Table 14. Appraisal of cross-sectional studies: RESULTS, DISCUSSION, OTHER CONSIDERATIONS**

| Study | Were the basic data adequately described? | Does the response rate raise concerns about non-response bias? | If appropriate, was information about non-responders described? | Were the results internally consistent? | Were the results presented for all the analyses described in the methods? | Were the authors, discussions and conclusions justified in the results? | Were the limitations discussed? | Were there any funding sources or conflicts of interest that may affect the authors' interpretation of the results? | Was ethical approval or consent of the participants attained? |
| --- | --- | --- | --- | --- | --- | --- | --- | --- | --- |
| Abhilash et al, 2016 | Yes | Yes | No | Yes | Yes | Yes | Yes | Yes | Yes |
| Chacko et al,2008 | Yes | Unclear | NA | Yes | Yes | Yes | No | Unclear | Unclear |
| Chrispal et al,2010 | Yes | NA | NA | Yes | Yes | Yes | Yes | No | Yes |
| Kumar et al,2012 | Yes | NA | NA | Yes | Yes | Yes | Yes | Unclear | No |
| Premraj et al,2018 | Yes | NA | NA | Yes | No | Yes | No | No | Unclear |
| Varghese et al,2006 | Yes | NA | NA | Yes | Yes | Yes | Yes | Unclear | Yes |
| Varghese et al,2013 | Yes | NA | NA | Yes | Yes | Yes | No | No | Yes |
| Viswanathan et al, 2013 | Yes | NA | NA | NA | Yes | Yes | Yes | No | NA |
| Varghese et al,2014 | Yes | NA | NA | Yes | Yes | Yes | Yes | No | NA |
| Chrispal et al,2010* | Yes | NA | NA | Yes | Yes | Yes | Yes | No | Yes |
| Narayanan et al, 2002 | Yes | NA | NA | Yes | Yes | Yes | No | No | Yes |
| Palanivel et al, 2015 | Yes | NA | NA | Unclear | Yes | Yes | No | Unclear | No |
| Vivekanandan et al, 2010 | Yes | No | Yes | Yes | Yes | Yes | Yes | Unclear | Unclear |
| Stephen et al,2015 | Yes | No | Yes | Yes | Yes | Yes | No | No | Yes |
| Mathai et al,2003 | Yes | No | No | Yes | Yes | Yes | Yes | Unclear | Unclear |
| Narayanswamy et al, 2016 | Yes | NA | NA | Yes | Yes | Yes | No | No | Yes |
| Palanivel S, et al,2012 | Yes | NA | NA | Yes | Yes | Yes | No | No | Clear |
| Manjunath et al, 2017 | Yes | NA | NA | Yes | Yes | Yes | Yes | No | Yes |
| Muthiaiah et al, 2016 | Yes | No | Yes | Yes | Yes | Yes | Yes | No | Yes |
| Poovathingal et al, 2014 | Yes | NA | NA | Yes | No | Yes | No | Unclear | Yes |
| Razak et al, 2010 | Yes | NA | NA | Yes | Yes | Yes | No | Unclear | NA |
| Mathew et al,2006 | Yes | Yes | Yes | Yes | Yes | Yes | Yes | Unclear | Yes |
| Ramabhatta et al,2017 | Yes | NA | NA | Yes | Yes | Yes | No | No | Yes |
| Sahana et al,2015 | Yes | NA | NA | Yes | Yes | Yes | Yes | No | Yes |
| Muddaiah et al,2006 | Yes | Yes | NA | Yes | Yes | Yes | No | Unclear | NA |
| Subbalaxmi et al,2014 | Yes | NA | NA | Yes | Yes | Yes | No | Unclear | NA |

| Very low risk of bias (1-8) | Low risk of bias (9-17) | Moderate risk of bias (18-22) | High risk of bias (23-26) |
| --- | --- | --- | --- |
| Abhilash et al,2016 | Varghese et al,2006 | Chacko et al,2008 | Kumar et al,2012 |
| Chrispal et al,2010 | Varghese et al,2013 | Premraj et al,2018 | Palanivel H et al,2015 |
| Viswanathan et al,2013 | Narayanan et al,2002 | Vivekanandan et al,2010 | Mathai et al,2003 |
| Varghese et al,2014 | Stephen et al,2015 | Poovathingal et al,2014 | Razak et al,2010 |
| Chrispal et al,2010* | Narayanaswamy et al,2016 | Subbalaxmi et al,2014 |  |
| Manjunath et al,2017 | Palanivel S et al,2012 |  |  |
| Muthaiah et al,2016 | Mathew et al,2006 |  |  |
| Sahana et al,2015 | Ramabhatta et al,2017 |  |  |
|  | Mudhaiah et al,2006 |  |  |

**Table 15. Risk of bias in cross-sectional studies**

**Quality assessment and risk of bias tool for case-control studies, case series and case reports, Joanna Briggs Institute(JBI) Reviewer’s Manual (27)**

**Table 16.** **Case-control study appraisal tool**

| S No. | Question | Yes/No/Unclear/Not applicable (NA) |
| --- | --- | --- |
| 1. | Were the groups comparable other than the presence of disease in cases or the absence of disease in controls? |  |
| 2. | Were cases and controls appropriately matched? |  |
| 3. | Were the same criteria used for cases and controls |  |
| 4. | Was exposure measured in a standard reliable way? |  |
| 5. | Was exposure measured in the same way for cases and controls? |  |
| 6. | Were confounding factors identified? |  |
| 7. | Were strategies to deal with confounding factors stated? |  |
| 8. | Were outcomes assessed for cases and controls in a standard, valid reliable way? |  |
| 9. | Was the exposure period long enough to be meaningful? |  |
| 10. | Was appropriate statistical analysis used? |  |

**Table 17.** **Case series appraisal tool**

| S No. | Question | Yes/No/Unclear/Not applicable (NA) |
| --- | --- | --- |
| 1. | Were there clear criteria for inclusion in the case series? |  |
| 2. | Was the condition measured in a standard, reliable way for all participants included in the case series? |  |
| 3. | Were valid methods used for identification of the condition for all the particpiants included in case series? |  |
| 4. | Did the case series have consecutive inclusion of participants? |  |
| 5. | Did the case series have complete inclusion of the participants? |  |
| 6. | Was there clear reporting on the demographics of the participants of the study? |  |
| 7. | Was there clear reporting of the clinical information of participants of the study? |  |
| 8. | Were the outcomes or follow up results of the cases reported? |  |
| 9. | Was there clear reporting of the presenting sites/clinics demographic information? |  |
| 10. | Was statistical analysis appropriate? |  |

**Table 18. Case reports appraisal tool**

| S No. | Question | Yes/No/Unclear/Not applicable (NA) |
| --- | --- | --- |
| 1. | Were the patient's demographic features clearly described? |  |
| 2. | Was the patient's history clearly described and presented as a timeline? |  |
| 3. | Was the current clinical condition of the patient on presentation described? |  |
| 4. | Were the diagnostic tests or assessment methods and the results clearly described? |  |
| 5. | Were the interventions or treatment procedures clearly described? |  |
| 6. | Was the post intervention clinical condition clearly described? |  |
| 7. | Were adverse events or unanticipated events identified and described? |  |
| 8. | Does the case report provide take away reports? |  |

**Quality assessment of individual studies (case control, case series, case reports) using JBI tool**

**Table 19.** **Quality assessment: Case-control study**

| S No. | Question | Basheer et al, 2016 |
| --- | --- | --- |
| 1. | Were the groups comparable other than the presence of disease in cases or the absence of disease in controls? | Yes |
| 2. | Were cases and controls appropriately matched? | Yes |
| 3. | Were the same criteria used for cases and controls | Yes |
| 4. | Was exposure measured in a standard reliable way? | Yes |
| 5. | Was exposure measured in the same way for cases and controls? | Yes |
| 6. | Were confounding factors identified? | Yes |
| 7. | Were strategies to deal with confounding factors stated? | No |
| 8. | Were outcomes assessed for cases and controls in a standard, valid reliable way? | Yes |
| 9. | Was the exposure period long enough to be meaningful? | NA |
| 10. | Was appropriate statistical analysis used? | Yes |

**Table 20.** **Quality assessment: Case series**

| S No. | Question | Kumar et al,2008 | Katoch et al,2016 | Saifudheen et al,2012 | Prasannan et al,2017 |
| --- | --- | --- | --- | --- | --- |
| 1. | Were there clear criteria for inclusion in the case series? | Yes | Yes | Yes | Yes |
| 2. | Was the condition measured in a standard, reliable way for all participants included in the case series? | Yes | Yes | Yes | Yes |
| 3. | Were valid methods used for identification of the condition for all the participants included in case series? | Yes | Yes | Yes | Yes |
| 4. | Did the case series have consecutive inclusion of participants? | Unclear | Yes | Unclear | Unclear |
| 5. | Did the case series have complete inclusion of the participants? | Unclear | Yes | Yes | Unclear |
| 6. | Was there clear reporting on the demographics of the participants of the study? | No | Incomplete | Yes | Incomplete |
| 7. | Was there clear reporting of the clinical information of participants of the study? | Yes | Yes | Yes | Yes |
| 8. | Were the outcomes or follow up results of the cases reported? | Yes | Yes | Yes | Yes |
| 9. | Was there clear reporting of the presenting sites/clinics demographic information? | No | Yes | Yes | No |
| 10. | Was statistical analysis appropriate? | NA | NA | NA | NA |

**Table 21.** **Quality assessment: Case reports**

| S No. | Question | Manicka-m et al,2014 | Chand-y et al,2009 | Devarajan et al,2012 | Thangaratha-m et al,2006 | Bhat et al,2015 | Jagdishkuma-r et al,2016 | Kakaraparth-i et al,2014 | Madi et al,2014 | Sitalakshm-i et al,2005 |
| --- | --- | --- | --- | --- | --- | --- | --- | --- | --- | --- |
| 1. | Were the patient's demographic features clearly described? | No | Yes | Incomplete | Yes | Incomplete | Incomplete | No | Incomplete | Yes |
| 2. | Was the patient's history clearly described and presented as a timeline? | Yes | Yes | Yes | Yes | Yes | Yes | No | Yes | Yes |
| 3. | Was the current clinical condition of the patient on presentation described? | Yes | Yes | Yes | Yes | Yes | Yes | Yes | Yes | Yes |
| 4. | Were the diagnostic tests or assessment methods and the results clearly described? | Yes | Yes | Incomplete | Yes | Yes | Yes | Yes | Yes | Yes |
| 5. | Were the interventions or treatment procedures clearly described? | Yes | Yes | Yes | Yes | Yes | Yes | Yes | Yes | Yes |
| 6. | Was the post intervention clinical condition clearly described? | Yes | Yes | Yes | Yes | Yes | Yes | No | Yes | Yes |
| 7. | Were adverse events or unanticipated events identified and described? | NA | Yes | Yes | Yes | Yes | Yes | No | Yes | Yes |
| 8. | Does the case report provide take away reports? | Yes | Yes | Yes | Yes | Yes | Yes | Unclear | Yes | Yes |

## **Selected results of descriptive and analytical statistics of studies**

1. Abhilash et al,2016

**Table 22.** **Lab profile of specific pathogen associated AFI**

| **Scrub typhus (ST) Laboratory profile compared with AFI due to other causes** | | | | | | | | | | |
| --- | --- | --- | --- | --- | --- | --- | --- | --- | --- | --- |
| **Total ST patients (N=451)** | | **Number of ST patients(n) with specific lab parameter** | | **Other AFI (807)** | | **Adjusted OR with other AFI** | | **95% CI** | | **P value** |
| TLC>10000cells/mm^3^  n (%) | | 193(42.89%) | | 111(13.79%) | | 2.31 | | 1.64-3.24 | | <0.001 |
| Total bilirubin  Mean (SD) | | 1.89(2.62) | | 1.32(2.37) | | 0.95 | | 0.89-1.02 | | <0.001 |
| Total albumin<3.5g%  n (%) | | 316(70.85%) | | 246(31.18) | | 2.32 | | 1.68-3.2 | | <0.001 |
| ALP  Mean (SD) | | 166.79(108.32) | | 107.02(82.79) | | 1.003 | | 1.001-1.005 | | <0.001 |
| **Dengue Laboratory profile compared with AFI due to other causes** | | | | | | | | | | |
| **Total Dengue patients (N=386)** | **Number of dengue patients with specific lab parameter** | | **0therAFI N=872** | | **Adjusted OR with other AFI** | | **95% CI** | | **P value** | |
| TLC<10000 cells/mm^3^  **n (%)** | 349(90.89%) | | 602(69.12%) | | 2.37 | | 1.56-3.59 | | <0.001 | |
| PC < 150000 cells/mm^3^  **n (%)** | 329(85.45%) | | 626(72.04%) | | 2.09 | | 1.47-2.98 | | <0.001 | |
| **Malaria Laboratory profile compared with AFI due to other causes** | | | | | | | | | | |
| **Total malaria patients (N=131)** | **Number of malaria patients with specific lab parameter** | | **Total other AFI=872** | | **Adjusted OR** | | **95% CI** | | **P value** | |
| PC<150000 cells/mm^3^  n (%) | 119(92.25%) | | 836(74.31%) | | 3.77 | | 1.92-7.39 | | <0.001 | |
| Total bilirubin  Mean (SD) | 3.03(3.85) | | 1.36(2.20) | | 1.19 | | 1.11-1.26 | | <0.001 | |
| SGPT (3 times ULN) n (%) | 1(0.79%) | | 179(16.01%) | | 0.02 | | 0.002-0.18 | | <0.001 | |
| **Enteric fever Laboratory profile compared with AFI due to other causes** | | | | | | | | | | |
| **Total enteric fever patients n=47** | **Number of enteric fever patients with specific lab parameter** | | **Other AFI** | | **Adjusted OR** | | **CI** | | **P value** | |
| PC<150000 cells/mm^3^ | 21(44.68%) | | 934(77.38%) | | 0.27 | | 0.14-0.51 | | <0.001 | |

1. Chacko et al, 2008

**Table 23. Lab profile of Dengue: Dengue Shock Syndrome (DSS) compared with non DSS**

| **Lab parameter** | **Total dengue patients tested n[N] (%)** | **DSS n=34** | **Non DSS n=39** | **p** |
| --- | --- | --- | --- | --- |
| SGPT>40IU/l | 31[69] (44.62%) | 20[30] (66.67%) | 11[39] (28.21%) | 0.0174 |
| Na^[[1]](#footnote-1)^<130 meq/l | 12[62] (19.05%) | 8[31] (24.8%) | 4[32] (12.5%) | <0.001 |
| HCO_3_^[[2]](#footnote-2)^<18mmol/l | 9[62] (14.52%) | 8[30] (26.67%) | 1[32] (3.13%) | 0.008 |
| Hb>12g% | 33(45.2%) | 22(64.71%) | 11(28.21%) | 0.0005 |
| Hct^[[3]](#footnote-3)^>35% | 49(67.1%) | 28(82.35%) | 21(53.85%) | 0.0098 |
| TLC<4000/cmm^3^ | 33(45.2%) | 25(73.52%) | 8(20.51%) | <0.0001 |
| PC<100000 cmm^3^ | 26(35.6%) | 20(58.82%) | 6(15.39%) | 0.0001 |
| INR^[[4]](#footnote-4)^>1.5 | 6[29] (20.69%) | 4[17] (23.53%) | 2[12] (16.67%) | 0.029 |

1. Chrispal et al,2010

**Table 24. Lab profile of specific pathogen associated AFI**

| **ST Laboratory profile compared with AFI due to other causes** | | | | | |
| --- | --- | --- | --- | --- | --- |
| **Lab parameter** | **Number of patients with ST (189)** | **Number of patients with other AFI (177)** | **P value** | **Adjusted OR** | **95% CI** |
| Hb(g%)  Mean (SD) | 12(2.3) | 11.5(2.9) | 0.045 |  |  |
| TLC>11500cells/cmm^3^  Number of patients(n) | 70 | 42 | 0.006 | 1.35 | 0.8-2.26 |
| Neutrophils %  Mean (SD) | 74.14(13.6) | 67.73(16.4) | <0.001 |  |  |
| Total bilirubin(mg%)  Mean (SD) | 2.15(2.4) | 5.68(9.2) | <0.001 |  |  |
| Creatinine mg%  Mean (SD) | 1.26(1.1) | 1.74(1.9) | 0.004 |  |  |
| S ALP(IU/L)  Mean (SD) | 177.96(127) | 128(86.8) | <0.001 |  |  |
| Elevated S ALT (IU/L) (45–200IU/L) | 134 | 81 | <0.001 | 3.78 | 2.29-6.21 |
| Serum albumin(<3.5g%)  Number of patients(n) | 160 | 127 | 0.002 | 1.76 | 0.97-3.19 |
| ARDS^[[5]](#footnote-5)^  Number of patients(n) | 47 | 12 | <0.001 | 6.56 | 3.12-13.80 |
| Aseptic meningitis  Number of patients(n) | 47 | 12 | <0.001 | 3.65 | 1.91-6.95 |
| **Malaria Laboratory profile compared with AFI due to other causes** | | | | | |
| **Lab parameter** | **Number of patients with malaria (68)** | **Number of patients with other AFI (298)** | **P value** | **Adjusted OR** | **95% CI** |
| Hb g% Mean (SD) | 10.3(3) | 12.1(2.4) | <0.001 |  |  |
| TLC (<11,500)  Number of patients(n) | 58 | 192 | 0.001 | 2.59 | 1.05-6.37 |
| Total bilirubin(>2g%)  Number of patients(n) | 53 | 86 | <0.001 | 9.40 | 4.11-21.48 |
| Total protein(g%)  Mean (SD) | 5.94(0.9) | 6.36(1.1) | 0.002 |  |  |
| S ALP(U/L)  Mean (SD) | 108.09(51.3) | 164(9119.5) | <0.001 |  |  |
| S creatinine>1.4mg%  Number of patients (n) | 26 | 60 | 0.002 | 9.96 | 4.15-23.88 |
| Thrombocytopenia  <50000cells/mm^3^  Number of patients (n) | 48 | 81 | <0.001 | 4.65 | 1.68-12.86 |
| S ALT<100U/L  Number of patients (n) | 51 | 117 | <0.001 | 17.02 | 6.74-42.97 |
| **Dengue Laboratory profile compared with AFI due to other causes** | | | | | |
| **Lab parameter** | **Number of patients with Dengue (28)** | **Number of patients with other AFI (270)** | **P value** | **Adjusted OR** | **95% CI** |
| TLC<11500cells/mm^3^  Number of patients (n) | 22 | 228 | 0.224 | 2.92 | 0.92-9.26 |
| Neutrophils (%)  Mean (SD) | 61.89(15.6) | 71.8(15.1) | 0.001 |  |  |
| Lymphocytes Mean (SD) | 27.75(14.1) | 19.7(13) | 0.002 |  |  |
| Thrombocytopenia  <50000cells/cmm^3^  Number of patients (n) | 18 | 111 | 0.001 | 2.75 | 1.07-7.08 |
| S AST>500 IU/L  Number of patients (n) | 12 | 15 | <0.001 | 13.42 | 4.69-38.36 |
| Serum albumin Mean (SD) | 3.27(0.9) | 2.86(0.7) | 0.002 |  |  |
| **Enteric fever Laboratory profile compared with AFI due to other causes** | | | | | |
| **Lab parameter** | **Number of patients with enteric fever (32)** | **Patients with other AFI (266)** | **P value** | **Adjusted OR** | **95% CI** |
| TLC<7500cell/cmm^3^  Number of patients (n) | 20 | 124 | 0.005 | 2.823 | 1.334-5.971 |
| PC>130000cells/cmm^3^  Number of patients (n) | 18 | 102 | 0.002 | 3.136 | 1.481-6.641 |
| Serum creatinine  mean (SD) | 1.02(0.5) | 1.54(1.6) | <0.001 |  |  |
| Total bilirubin  mean (SD) | 2.02(3.4) | 4.02(7) | 0.01 |  |  |
| Total protein  mean (SD) | 6.99(0.9) | 6.22(1) | <0.001 |  |  |
| Serum albumin  mean (SD) | 3.33(0.7) | 2.84(0.7) | <0.001 |  |  |

1. Kumar et al,2012

**Table 25****. Lab profile of ST patients**

| **Lab parameter** | **Number of patients with ST(n)** | **Percentage (%)** |
| --- | --- | --- |
| Raised Creatinine | 7 | 20% |
| Decreased albumin | 19 | 54% |
| Increased AST/ALT | 11 | 31% |
| Raised ALP | 10 | 29% |
| Bilirubin>1.2 mg/dl | 3 | 9% |
| Decreased Na | 6 | 17% |
| Raised CPKMB | 12 | 34% |
| Albuminuria | 1 | 3% |
| TLC<4000 cells/mm^3^ | 1 | 3% |
| TLC 4000-11000 cells/mm^3^ | 22 | 63% |
| >11000 cells/mm^3^ | 13 | 37% |
| Platelets>150000 cells/mm^3^ | 11 | 31% |
| 100000-150000 cells/mm^3^ | 14 | 40% |
| <100000 cells/mm^3^ | 11 | 31% |
| **WFT titers for ST diagnosis** | | |
| OX-K 1:80 | 3 | 9% |
| OX-K 1:160 | 14 | 40% |
| OX-K 1:320 | 18 | 51% |

1. Premraj et al,2018

**Table 26. Lab profile of ST cases**

| **Lab parameter** | **Mean (SD)** |
| --- | --- |
| Hb | 11.38(2.3) |
| TLC | 9.6(3.5) |
| PC | 143(74) |
| Serum creatinine | 1.04(0.38) |
| AST | 111(96) |
| ALT | 107(84) |
| Serum ALP | 141(76) |

Notes: The study did not show significant correlation with leukocytosis, hypoalbuminemia, anemia, hepatic dysfunction.

Thrombocytopenia noted in 52% of patients.

Leukocytosis noted in 40% patients

Hyponatremia noted in 62% patients showing serum Na of < 130 meq/l.

1. Varghese et al,2006

**Table 27. Lab profile of ST patients**

Total number of ST patients=50

| **Lab parameter** | **Percentage of patients of ST with abnormality** |
| --- | --- |
| Abnormal LFT | 90% |
| PC<100000/mm^3^ | 62.5% |

**Table 28. Significant findings by univariate analysis of ST patients compared with ST negative AFI controls (negative for IgM)**

| **Lab parameter** | **Total number of ST n=50** | **AFI with ST IgM negative**  **n=16(controls)** | **P value** |
| --- | --- | --- | --- |
| Transaminase level> 2*Normal | 36(90%)  (n=40) | 7(50%)  (n=14) | 0.004 |
| Bilirubin level>1.5mg% | 12(30%)  (n=40) | 9(64.3%)  (n=14) | 0.02 |
| Creatinine>1.4mg% | 5(12.2%)  (n=41) | 3(20%)  (n=15) | 0.66 |

**Table 29. Predictors of mortality**

| **Lab parameter** | **Expired n=7** | **Alive n=43** | **RR(CI)** | **P value** |
| --- | --- | --- | --- | --- |
| Bilirubin>1.5mg% | 5(71.4%) | 7(21.2%) | 9.28(1.48-58.5) | 0.02 |
| Creatinine>1.4mg% | 4(57.1%) | 1(2.9%) n=34 | 43.9(3.65-530.5) | 0.003 |

Note: Predictive values and specificities of combined tests like raised transaminases, thrombocytopenia and raised TLC are about 80% and depend on the prevalence of ST. Case fatality rate=14%

1. Varghese et al,2013

**Table 30. Lab parameters of ST: Comparison of patients alive versus those dead suffering from ST**

| **Lab parameter** | **Alive n=142(92.2%)** | **Dead n=12(7.8%)** | **P value** |
| --- | --- | --- | --- |
| Hb (g%) | 12(1.7) | 12.5(1.9) | 0.36 |
| TLC*10^9^ /l  Median (IQR) | 10.4(1.2-36.6) | 14.4(7.7-36) | 0.001 |
| PC*10^9^/l  Median (IQR) | 79(3-368) | 38(15-98) | 0.03 |
| Serum creatinine Median (IQR) | 1.1(0.5-6.6) | 2.2(0.6-8.6) | 0.001 |
| CPK Median (IQR) | 94(21-3010) | 261(82-2172) | 0.03 |
| Abnormal CXR Median (IQR) | 83(58.5%) | 11(91.7%) | 0.03 |

**Table 31. Predictors of mortality for ST**

| **Univariate analysis: Prediction of mortality factors associated with mortality** | | | | | | |
| --- | --- | --- | --- | --- | --- | --- |
| **Complication** | **Total n (%)** | **Alive n (%)** | **Dead n (%)** | **P value** | **RR** | **95% CI** |
| ARDS | 67(43.5%) | 58(40.8%) | 9(75%) | 0.03 | 4.3 | (1.1-16.7) |
| Hepatitis  Bilirubin> 2.5mg/dl | 99(64.2%) | 91(64.1%) | 8(66.7%) | 0.29 | 1.9 | (0.56-6.3) |
| Renal failure  Creatinine> 2.5mg/dl  Number of patients n(%) | 20(12.9%) | 15(10.6%) | 5(41.7%) | 0.005 | 6.04 | (1.7-21.4) |

| **Lab parameters as predictors of mortality (by multivariate analysis)** | | | | | |
| --- | --- | --- | --- | --- | --- |
| Lab parameter | Alive n=142(92.2%) | Dead n=12(7.8%) | RR | 95% CI | P value |
| TLC *10^9^/l | 10.4(1.2-36.6) | 14.4(7.7-36) | 0.99 |  | 0.01 |
| Bilirubin>2.5mg/dl | 36(25.4%) | 5(41.7%) | 1.9 | 0.57-6.4 | 0.29 |
| Creatinine Median (IQR) | 1.1(0.5-6.6) | 2.2(0.6-8.6) | 1.75 | 1.25-2.47 | 0.001 |
| creatinine>2.5mg/dl | 15(10.6%) | 5(41.7%) | 6.04 | 1.6-13.6 | 0.005 |

Case fatality rate of ST patients in this study=7.8%

1. Viswanathan et al,2013

**Table 32. Lab parameters of ST patients without meningitis versus with meningitis**

| **Lab parameters** | **Number of patients with ST without meningitis**  **N=48** | **Number of patients with ST with meningitis**  **N=17** | **P value** |
| --- | --- | --- | --- |
| Urea mmol/l Mean (SD) | 9.97(7.91) | 17.08(12.90) | 0.01 |
| Urea>7mmol/l  Number of patient n (%) | 25(52.1%) | 14(82.4%) | 0.029 |
| TLC*10/l | 8.32(2.91) | 13.02(7.88) | 0.028 |
| Normal CXR  Number of patient n (%) | 30(62.5%) | 15(88.2%) | 0.048 |

1. Varghese et al,2014

**Table 33. Lab parameters of ST with Multiple Organ Dysfunction Syndrome (MODS) versus ST without MODS**

| **Patient characteristics** | **Multiorgan dysfunction present**  **N=212(34%)** | **Multiorgan dysfunction absent**  **N=411(66%)** |
| --- | --- | --- |
| Age Mean (SD) | 45.6(14.8) | 44(15.8) |
| Sex Male/female | 96/116 | 204/207 |
| Occupation: Agriculture | 87(47%) | 176(42.8%) |
| Occupation: Others | 125(58.9%) | 235(57.1%) |
| Hb g/dl Mean (SD) | 11.8(2.5) | 11.8(2.2) |
| WBC *10^9^/l median (IQR) | 11.65(1.20-50) | 9.8(1.9-36.60) |
| PC*10^9^/l | 51(5-343) | 95(3-529) |
| Bilirubin mg/dl Median (IQR) | 2.6(0.3-30.9) | 0.8(0.2-14.4) |
| Total protein Mean (SD) | 5.8(0.7) | 6.5(0.8) |
| Albumin g/dl Mean (SD) | 2.3(0.5) | 2.8(0.6) |
| AST IU/l Median (IQR) | 156(14-2698) | 116(12-1839) |
| ALT IU/l Median (IQR) | 83(12-1775) | 80(10-753) |
| ALP IU/l median (IQR) | 187(58-975) | 122(24-715) |
| S creatinine mg/dl Median (IQR) | 1.8(0.6-12.5) | 1(0.4-5.7) |
| CPK IU/l Median (IQR) | 184(21-22234) | 87(20-140500) |
| Case fatality n (%) | 53(25) | 3(0.7) |

**Table 34. Predictors of mortality of ST: Lab parameters (Univariate analysis)**

| **Lab parameter** | **Dead n=56(8.9%)** | **Alive n=567(91%)** | **RR(CI)** | **P value** |
| --- | --- | --- | --- | --- |
| TLC*10^9^/l Median (IQR) | 15.5178(2.200-42.100) | 10.785(1.200-50.000) | 1(1-1) | <0.001 |
| PC*10^9^/l Median (IQR) | 65.268(6.000-333.000) | 100.051(3.00-529.00) | 1(1-1) | 0.002 |
| AST IU/L Median (IQR) | 241.9(31-2698) | 171.1(12-1850) | 1.001(1-1.002) | 0.02 |
| Bilirubin> 2.5mg/dl  Number of patients n (%) | 33(58.9) | 179(31.6) | 3.1(1.7-5.4) | <0.001 |
| creatinine>2.5/dl  Number of patients n (%) | 32(57.1%) | 80(14.1%) | 8.1(4.5-14.5) | <0.001 |
| CNS dysfunction  Number of patients n (%) | 32(57.1%) | 39(6.9%) | 18.1(9.6-33.6) | <0.001 |

Notes:

Common Lab findings- Raised transaminases (87%), thrombocytopenia (79%), leukocytosis (46%). CPK levels high in MODS as compared to group with no MODS (mean value1336 vs 135IU/L, p<0.001). Overall case fatality rate=9%Case fatality higher in group with MODS compared with no MODS (25%vs 0.7%, p<0.001)

1. Chrispal et al,2010*

**Table 35. Lab parameters of ST**

| **Lab parameter** | **ST patients who died n=23**  **Mean (SD)** | **ST patients who survived n=166 Mean (SD)** | **P value** |
| --- | --- | --- | --- |
| TLC (cells/mm^3^) | 14767.83(6986.3) | 10558.43(5016.1) | <0.001 |
| DLC^[[6]](#footnote-6)^ Band forms (%) | 7.5% (10.9) | 2.6% (3.9) | 0.045 |
| HCO_3_(mmol/l) | 14.2(14.8) | 19.2(4.2) | <0.001 |
| Albumin (g%) | 2.5(0.4) | 2.8(0.7) | 0.002 |
| AST(IU/L) | 218.4(161.2) | 155.9(133.5) | 0.042 |

**Table 36. Predictors of mortality in ST**

|  | **Dead n=23** | **Survivors n=166** | **P value** | **OR** | **95%CI** |
| --- | --- | --- | --- | --- | --- |
| Metabolic Acidosis | 18% | 37% | <0.001 | 6.1 | 1.773-21.272 |
| ARDS | 14% | 33% | <0.001 | 3.6 | 1.183-10.741 |
| RF^[[7]](#footnote-7)^ | 11% | 26% | 0.001 | 1 | 0.296-3.681 |

Notes: Definitions

ARDS=bilateral pulmonary infiltrates on CXR, peak flow rate <200, normal CVP^[[8]](#footnote-8)^

RF= creatinine>1.4mg%

Venous Bicarbonate<17mmol/l (Metabolic acidosis)

1. Narayanan et al,2002

**Table 37. Lab parameters in Dengue: Variation according to severity of Dengue**

| **Parameter** | **Dengue infection** | **DF** | **DFB** | **DHF** | **DSS** | **P value** |
| --- | --- | --- | --- | --- | --- | --- |
| **Number of cases** | 59 | 20 | 23 | 11 | 5 | - |
| **Mean age(years) (SD)** | 6.8(3.2) | 7.1(3) | 6.4(3.3) | 7.8(3.5) | 4.6(1.8) | - |
| **Investigations** | | | | | | |
| **Hb(g/dl) Mean (SD)** | 10.8(1.1) | 10.8(0.6) | 11.1(0.7) | 10.3(1.9) | 11.4(1.5) | 0.35 |
| **Hct**  **Mean (SD)** | 33.2(3.3) | 32.2(2) | 32.1(2.4) | 35.2(5.6) | 37.6(2.9) | 0.0002 |
| **PC cells/mm^3^**  **mean** | 89559 | 96550 | 108782 | 60909 | 36200 | 0.008 |
| **PC 50001-100000/mm^3^**  **Number of patients, n (%)** | 31 | 13 | 11 | 6 | 1 | 0.0041 |
| **AST>50**  **Number of patients, n (%)** | 42(71.9) | 16(80%) | 14(60.9%) | 9(81.8%) | 2(66) n=3 | 0.39 |
| **ALT>50IU/L**  **Number of patients, n (%)** | 34(59.7%) | 13(65%) | 12(52.1%) | 7(63.6%) | 2(66%) n-3 | 0.69 |
| **ALP>200IU/L**  **Number of patients, n (%)** | 24(42.1%) | 11(55%) | 5(21.7%) | 5(45.4%) | 2(66%) n=3 | 0.07 |

Notes: Mean Hematocrit was significantly higher in DHF and DSS groups(p=0.0002)

PC was significantly lower in DHF and DSS groups (p=0.0041)

1. Palanivel H, et al,2012

Study on the clinical and lab profile of Dengue classified according to WHO criteria

Notes on the study:

Minimum age at which Dengue NS1 detected=6-month-old child

Out of all the children serologically positive for dengue:

NS1 antigen positive=85% (95% CI=78,92)

IgM positive=6% (95%, CI=1,11)

IgG positive=3% (95% CI=0,6)

NS1Ag and IgM positive=4.9%

NS1 Ag, IgM and IgG positive=0.6%

Notes: NS1 antigen detected as early as the first day of fever with detection limit up to 10 days of fever and a positivity rate of 93%

Peak period of detection ranged from 3^rd^ to 6^th^ day (CI=88,98) and highest on the 4^th^ day (28%) found to be significant (p=0.005). NS1 is an early diagnostic marker

IgM not detectable prior to day 3 of the illness

IgM detectable by the 4^th^ day of fever with positivity rate of 11 %(CI=5,7)

IgM alone associated with thrombocytopenia in 67% of patients

IgM with NS1 antigen associated with thrombocytopenia in 78%

NS1 antigen associated with thrombocytopenia in 49%

In this study, thrombocytopenia noted in 50% patients. Among these 24 required platelet transfusions.

21/24 of these patients were NS1 antigen positive, so can be considered a marker of low platelet.

1. Vivekanandan et al,2010

**Table 38. Lab parameters in ST patients**

| **Lab parameter** | **Number of patients n (%)** |
| --- | --- |
| Renal impairment(>1.5mg/dl) | 6(12%) |
| Bilirubin>1.2mg/dl | 8(16%) |
| Thrombocytopenia (<1 lakh/mm^3^) | 5(10%) |
| TLC<4000/mm^3^ | 1(2%) |
| TLC 4000-11000/mm^3^ | 34(68%) |
| TLC >11000/mm^3^ | 15(30%) |
| PC <1.5 lakh/mm^3^ | 8/46(17.3%) |
| PC<1 lakh/mm^3^ | 5/46(10.8%) |
| Raised SGOT/SGPT | 47/49(95.9%) |
| Raised ALP | 14/28(50%) |
| Albumin<3g/dl | 21/24(87.5%) |
| Albuminuria | 33/50(66%) |
| Raised creatinine(>1.5mg%) | 6/46(13%) |
| Raised Bilirubin(>1.2mg/dl) | 8/39(20.5%) |
| **WFT** | **Number of patients n** |
| 1:80 | 5 |
| 1:160 | 13 |
| 1:320 | 21 |
| Total | 39/50(78%) |

Notes: seasonal variation in cooler months from September to April

1. Stephen et al,2015

**Table 39. Lab parameters of ST**

| **Lab parameter** | **Number of patients n (%)** | **Notes** |
| --- | --- | --- |
| Thrombocytopenia <150000/mm^3^ | 13/28(46.43%) 1 child and 12 adults |  |
| Leukocytosis>11000/mm3 | 5/7 children (71.4%) 4/18 adults (22.22%) 9/25 total (36%) |  |
| Liver enzymes and significant rise  >twice normal | 1 child and 14 adults |  |
| ST RICT | 24 positives on ST RICT, IgM and IgG | 4 patients false positive on RICT |
| ST IgG ELISA | 23 had IgG antibodies in acute and/or convalescent sera |  |
| WFT | 21 positives on WFT | OXK ranged from 1:40 to 1:10240 |

**Table 40. Comparison with ST IgM ELISA as a reference standard**

| **Test** | **sensitivity** | **specificity** | **PPV** | **NPV** |
| --- | --- | --- | --- | --- |
| ST RICT | 91.67% | 90.48% | 91.67% | 90.48% |
| WFT | 83.33% | 95.24% | 95.24% | 83.33% |

1. Mathai et al,2003

**Table 41. Lab parameters of ST**

| **Lab parameter** | **Number of patients** | **Number of patients tested** |
| --- | --- | --- |
| TLC>11000/mm^3^ | 14(54%) | n=26 |
| PC<100000/mm^3^ | 9(43%) | n=21 |
| Transaminase level>twice normal | 22(88%) | n=25 |
| Bilirubin level>25micromol/l | 7(29%) | n=24 |
| Creatinine>120micromol/l | 8(37%) | n=24 |
| Abnormal CXR | 9(37%) | n=24 |

Notes: 5 pregnant women with ST, 4 had perinatal deaths and 1 had a preterm baby.

17 patients received Doxycycline showed fever defervescence in 1-3 days.

2 patients brought late to hospital with severe jaundice and died within 48 hours of admission.

1 patient developed rashes and gangrene and sepsis and died.

Overall mortality was 3(11.1%).

1. Narayanaswamy et al,2016

**Table 42. Lab profile of ST patients**

Total number of patients with ST=117

| **Lab parameter** | **Number of ST patients with deranged parameter** | **percentage** |
| --- | --- | --- |
| Anemia | 66 | 56% |
| TLC<4000/mm^3^ | 12 | 10% |
| 4000-11000/mm^3^ | 47 | 40% |
| >11000/mm^3^ | 58 | 50% |
| DLC: Raised neutrophils | 44 | 38% |
| DLC: Raised Lymphocytes | 76 | 65% |
| DLC: Raised Monocytes | 55 | 47% |
| DLC: Eosinopenia | 33 | 28% |
| PC |  |  |
| >150000/mm^3^ | 69 | 59% |
| 100000-150000/mm^3^ | 30 | 25% |
| <50000/mm^3^ | 4 | 3% |
| SGOT | 56 | 48% |
| SGPT | 57 | 48% |
| Hypoalbuminemia | 46 | 40% |
| Hyponatremia | 47 | 40% |
| Raised creatinine | 6 | 5% |
| Bilirubin>1.2 mg% | 3 | 2.5% |
| Hematuria | 1 | <1% |
| Proteinuria | 28 | 24% |
| USG findings of polyserositis | 19 | 16% |

1. Palanivel S, et al,2012

**Table 43. Lab profile paediatric ST patients**

| **Lab parameter** | **Number** | **Percentage** |
| --- | --- | --- |
| Hb<11g% | 56 | 83.58% |
| Thrombocytopenia<100000/mm^3^ | 52 | 77.61% |
| Raised SGOT and SGPT | 43 | 64.17% |

1. Manjunath et al,2017

**Table 44. Lab parameters of Paediatric ST patients**

| **Lab parameter** | **Number** | **Percentage** |
| --- | --- | --- |
| Thrombocytopenia (<50000/mm^3^) | 18 | 66.7% |
| Anemia | 13 | 48.1% |
| TLC>10000/mm^3^ | 5 | 18.5% |
| TLC<4000/mm^3^ | 1 | 3.7% |
| AST>40 IU/L | 23 | 85.2% |
| ALT>40 IU/L | 22 | 81.5% |
| S Na<135 meq/l | 17 | 62.9% |
| S albumin<3.5 g/dl | 15 | 55.6% |
| Elevated S ALP | 5 | 18.5% |
| bilirubin>1.5 | 2 | 7.4% |

**Table 45. Complications of ST: Relevant Lab parameters**

| **Complication of ST** | **Number of patients, n (%)** |
| --- | --- |
| Hepatitis (AST and ALT>3 times ULN) | 4(14.8%) |
| Cardiac involvement | 4(14.8%) |
| CPK-MB>2 times ULN | 2(7.4%) |
| CPK-MB>4 times ULN | 2(7.4%) |

1. Muthaiah et al,2016

**Table 46. Lab profile in AFI patients: Survivors versus patients who died**

| **Lab parameter** | **Recovered n (%)** | **Died n (%)** |
| --- | --- | --- |
| PC on admission |  |  |
| >1lakh/mm^3^ | 14(83.4%) | 3(17.6%) |
| 50000-1 lakh/mm^3^ | 8(57.1%) | 6(42.9%) |
| <50000/mm^3^ | 32(72.7%) | 12(27.3%) |
| creatinine |  |  |
| < 1.4 mg% | 22(68.8%) | 10(31.3%) |
| >1.4 mg% | 32(75%) | 11(28%) |

1. Poovathingal et al,2014

**Table 47. Lab profile of patients with malaria**

| **Lab parameter** | **Number of patients with deranged lab parameter, n**  **Total number of malaria patients=183** | **percentage** |
| --- | --- | --- |
| Hb<5g% | 3 | 1.6% |
| TLC>15000/microL | 28 | 33.73% |
| PC<1 Lakh/microL | 128 | 69.9% |
| PlC<5000/microL | 5 | 2.7% |

**Table 48. Results of diagnostic tests for malaria**

| **Test result** | **Falcivax** | **MP QBC** | **PBS** |
| --- | --- | --- | --- |
| Positive | 161 | 165 | 178 |
| Negative | 10 | 18 | 5 |

**Table 49. Parasite index on malarial smear**

| **Parasite Index** | **Number of malaria cases, n** |
| --- | --- |
| <0.5 | 69 |
| 0.5-1 | 13 |
| 1-5 | 37 |
| 5-10 | 4 |
| >10 | 1 |

**Table 50. Parasite index and serum creatinine as predictors of mortality**

| **Death** | **Cases** | **Parasite Index Mean (SD)** | **Creatinine Mean (SD)** |
| --- | --- | --- | --- |
| Yes | 12 | 2.9(3.2) | 4.4(4.19) |
| No | 112 | 1(1.6) | 1.63(1.39) |

Notes: Correlation between creatinine and death was significant p<0.0001. Parasite index and death was significant with p<0.01

Severe cases had an average mean (SD) of ESR 60.10(42.30) compared to the rest of the cases=38.83(31.87).

93(50.8%) cases had severe malaria according to WHO criteria

Relationship between WHO severity and ESR was significant with p<0.003

**Table 51. ESR in cases of malaria**

| **ESR** | **Number of cases, n** |
| --- | --- |
| 10-50mm/h | 94 |
| 50-100mm/h | 40 |
| 100-150mm/h | 30 |

1. Razak et al, 2010

**Table 52. Lab parameters of ST**

Total number of ST patients= 29

| **Lab parameter** | **Number of patients with specific lab parameter** | **Percentage** |
| --- | --- | --- |
| Thrombocytopenia  <1.5lakh/mm^3^ | 18 | 62% |
| TLC>12000cells/mm^3^ | 11 | 37% |
| Anemia Hb<10g% | 7 | 24% |
| Bilirubin>3mg% | 10 | 34% |
| Albumin<3.5g/dl | 20 | 68% |
| ARF Creatinine>1.6mg% | 2 | 6% |
| Hypokalemia k+<3.5mg/dl | 6 | 20% |
| CPK raised 5 times above normal | 10 | 34% |
| CSF analysis lymphocyte predominance in all 3 patients | | |
| CXR ARDS | 3 | 10% |
| CXR pleural effusion | 2 | 6% |
| USG abdomen hepatosplenomegaly | 12 | 41% |
| Echo Myocarditis | 1 | 3% |
| WFT titers in diagnosed cases of ST | | |
| WFT Titer | Number of patients of ST | Percentage |
| 1:1280 | 2 | 6% |
| 1:640 | 8 | 27% |
| 1:320 | 9 | 31% |
| 1:160 | 10 | 34% |

1. Mathew et al,2006

**Table 53. Mean values of lab parameters in neuroleptospirosis patients, Lab parameters and number of cases showing specific lab parameters**

| **Lab parameter** | **Mean (SD)** |
| --- | --- |
| Hb g% | 12.5(1.9) |
| TLC cells/microL | 11288(4643) |
| Platelet count *10^5^ cells/microL | 1.3(0.98) |
| Blood urea levels mg% | 84.5(58.5) |
| Creatinine mg% | 1.8(1.3) |
| Bilirubin mg% | 3.5(2.5) |
| SGOT U/L | 524(1068) |
| SGPT U/L | 503(1453) |
| Mean CSF cell count cells/microL | 50.2(72) |
| **Lab parameters in Neuroleptospirosis** | |
| **Lab parameter** | **Number, n (%)** |
| Leucocytosis | 17/28(61%) |
| Neutrophilic leucocytosis | 16/17(96%) |
| PC range | 1.6-3.4* 10^5^cells/microL |
| Deranged RFT | 20(64.5%) |
| LFT deranged with increased bilirubin | 23/30(74%) |
| Lymphocytic pleocytosis (CSF) | 13/18(72%) |

Notes: The patients of the sample were divided into 2 groups A and B and compared based on CSF analysis

Survived group A=23 Succumbed group=8

CSF protein significantly higher in group B compared to group A, p<0.001

Group B mean CSF protein Mean (SD) = 183(73.2) mg%

Group A mean CSF protein Mean (SD) = 90(45.72) mg%

1. Ramabhatta et al,2017

**Table 54. Lab parameters of paediatric dengue patients**

| **Lab parameter** | **Number of children, n (%)** |
| --- | --- |
| <20000/mm^3^ | 36 |
| 21000-50000/mm^3^ | 204(35.9%) |
| 50000-100000/mm^3^ | 236(41.5%) |
| Hct>38 | 264(46%) |
| USG |  |
| Edema gall bladder | 65% |
| hepatosplenomegaly | 12.5% |
| Ascites | 71.6% |
| CXR | 168(29.5%) |
| LFT abnormal | 30.6% |
| PT^[[9]](#footnote-9)^ abnormal | 0.18% |
| APTT^[[10]](#footnote-10)^ abnormal | 0.7% |
| NS1 Antigen positive | 442(78%) |
| IgM positive | 90(15.8%) |
| IgG positive | 82(14.6%) |
| NS1 Antigen positive and no antibody | 363(64%) |

Notes:

Majority of patients with SD had abnormal LFT.

No correlation between platelets and bleeding manifestation.

IgG positive patients had more complications than IgM positive patients suggestive of the fact that secondary dengue is more severe than primary dengue.

Bleeding as a clinical manifestation among IgG positive showed statistical significance(p<0,05).

1. Sahana et al, 2015

**Table 55. Performance of specific and nonspecific diagnostic tests in dengue patients**

| **Test** | **Percentage of cases** |
| --- | --- |
| NS1 antigen positive | 66.7% |
| IgM ELISA positive | 29.6% |
| IgG ELISA positive (along with IgM or NS1 antigen positive) | 18.5% |
| NS1 positive within 5 days of illness onset | 88% out of the 66.7% (overall percentage of cases positive for NS1 antigen) |
| IgM positive | Within 3days to 15 days of illness |
| IgG positive | Within 2-15 days of illness |
| Anemia | 14.8% patients |
| Hemoconcentration>40  (mean PCV=37.5%) | 72.8% |
| Thrombocytopenia | 82.7% (6.2% out of 82.7% had PC<20000) |
| Bleeding manifestations (percentage of cases) | PC |
| 0 | <20000/mm^3^ |
| 33.3% | 20000-50000/mm^3^ |
| 23.1% | 50000-1 lakh/mm^3^ |

**Table 56. Logistic regression analysis of risk factors for severe dengue**

| **Factor** | **OR** | **95%CI** | **P value** |
| --- | --- | --- | --- |
| **LFT** | 17.836 | 2.563-124.072 | 0.004 |
| **Gall bladder** | 1.7 | 0.060-48.112 | 0.756 |

**Table 57. Lab parameters in various forms of Dengue**

| Lab parameter | D (39) | DW (22) | SD (20) |
| --- | --- | --- | --- |
| Hemoconcentration  >40 | 10(25.7%) | 9(40.9%) | 11(55%) |
| PC<1.5 lakh/mm^3^ | 27(69.3%) | 21(95.5%) | 19(95%) |
| Leucopenia | 16(41%) | 3(13.6%) | 9(45%) |
| LFT | 2(5.1%) | 9(40.9%) | 16(80%) |
| Coagulation profile(deranged) | 0 | 2(9.1%) | 6(30%) |
| USG Abdomen | 16(41%) | 20(90.9%) | 18(90%) |
| Gall bladder edema | 9(23.1%) | 17(77.3%) | 17(85%) |

Notes: USG abdomen: 66.7% cases with gall bladder wall thickening seen significantly high in severe dengue cases (SD)(p<0.001)

LFT abnormal in 33.3% of total patients whereas 80% of SD had abnormal LFT which is statistically significant(p<0.001)

PT and raised APTT=9.9% patients

Ascites 95% CI (2.026-1.934.986), p=0.0181, significantly associated with severe dengue.

PC<20000/mm^3^ patients transfused with platelets.

1. Muddaiah et al,2006

**Table 58. Lab profile of malaria patients**

| **Lab parameter** | **Number of patients (n)** | **Percentage** |
| --- | --- | --- |
| ESR | 104 | 53.7% |
| Anemia | 27 | 14.27% |
| Leucopenia | 60 | 31.57% |
| Thrombocytopenia | 16 | 8.42% |
| BUL | 21 | 14.21% |
| S creatinine | 14 | 11.57% |

**Table 59. Complications of malaria: Lab parameters**

| **Lab parameter** | **Number** | **Percentage** |
| --- | --- | --- |
| Abnormal LFT | 28 |  |
| Raised indirect and direct bilirubin | 28 | 13.68% |
| SGOT | 26 | 13.68% |
| SGPT | 25 | 13.15% |
| Urine analysis(proteinuria) | 16 | 8.42% |

Notes: A study of malaria. Sample size, N=314, 124 outpatients and 190 were inpatients

Inpatient analysis 190 cases, 153(80.5%) M and 37(19.5%) F

Notes: Seasonal variation: Number of admissions due to malaria increased from June onwards showing a similar pattern in outpatient data, increased from June to October.

186(98%) patients out of 190 improved and were discharged. 2 patients took DAMA and 2 patients died.

1. Subbalaxmi et al,2014

**Table 60. Lab parameters in ST patients**

| **Lab parameter** | **Number of patients** | **Percentage** |
| --- | --- | --- |
| Mean Hb | Mean=11.1g% |  |
| Leucopenia | 18 | 10.2% |
| Leukocytosis | 42 | 23.9% |
| PC<1 lakh/mm^3^ | 53 | 30.1% |
| Raised AST | 153 | 86.9% |
| Raised ALT | 136 | 77.3% |
| Raised creatinine | 49 | 27.8% |
| Raised ALP | 110 | 62.5% |
| Infiltrates on CXR | 46 | 26.1% |

1. Basheer et al,2016

**Table 61. Comparison of lab parameters of dengue and ST coinfection with dengue and ST cases alone**

| **Parameter** | **Number of dengue controls**  **n=18** | **Number of ST controls**  **n=18** | **Dengue and ST coinfection cases**  **n=6** | **P value** |
| --- | --- | --- | --- | --- |
| Hb g% Mean (SD) | 15.7(1.3) | 11(1.5) | 10.6(1.2) | <0.001 |
| TLC*10^9^/l Mean (SD) | 3.9(0.7) | 11.6(2.9) | 8.8(1) | <0.002 |
| Lowest PC*10^9^/l  Mean (SD) | 44.5(28.5) | 63.8(22.4) | 21.7(11.5) | 0.002 |
| Urea mg/dl  Mean (SD) | 18.2(7.4) | 41(17.3) | 31.8(7.2) | <0.001 |
| Creatinine mg/dl  Median (IQR) | 0.7(0.6-0.8) | 0.8(0.5-1.1) | 1.1(0.9-1.2) | 0.07 |
| Total bilirubin | 0.5(3) | 1.6(0.3) | 1.6(0.2) | <0.001 |
| APTT  Mean (SD) | 51(8.9) | 44.8(7.2) | 57.7(6) | 0.003 |

Notes: S albumin levels in coinfection versus dengue (3 vs 3.9 g/dl, p<0.001) was significant, but not with isolated ST (3 vs 2.9 g/dl, p=0.95)

**Table 62. Case reports: Results of lab investigations**

| **Study number** | **Study** | **Diagnosis** | **Patient characteristics** | **Specific test results** | **Nonspecific test results** | **Management** | **Conclusion** |
| --- | --- | --- | --- | --- | --- | --- | --- |
| 1 | Manickam et al,2014 | A case of pediatric typhus presenting with massive consolidation | 9-year-old female child | Malaria antigen=negative  Dengue serology=negative  BC=sterile  WFT titers>160 for OX-K antigen. Titers for OX-2 and OX-19 were negative  IgM ELISA for ST strongly positive | ESR=42mm/h  PC on 2 occasions=138 and 148(*10^9^cells/l)  CRP=57.44mg/l  USG= No abnormality | Doxycycline for 5 days with resolution of illness | Atypical presentation of ST |
| 2 | Chandy et al,2009 | Case report on Hanta virus infection as a cause of AFI | 46 year-old-male granary worker from Cudappah in AP | Acute phase sample positive for Hantavirus IgM and IgG and IFA and ELISA | Hb=5.9 g%  TLC=1900cells/mm^3^  PC=87000cells/mm^3^  Presence of band forms and giant platelets  APTT=44s  PT=15.1s  S creatinine=4.9 mg%  Urea=128 mg%  Urine analysis=Protienuria3+  Hematuria=15-20 RBCs/hpf  Presence of coarse fine granular casts  LFT=Hyperbilirubinemia=2 mg%  Hypoalbuminemia=2.7 g%  ALT=2.7 mg%  LDH=722 U/L  CPK=344 U/L  S phosphorus=7.4 | Supportive therapy given for the illness | A cause of AFI complicated by hepatorenal syndrome |
| 3 | Devarajan et al,2012 | A case of AFI with hematuria caused by ST | 55 year-old-male brought with AFI was initially treated for leptospirosis, did not respond to therapy and then was diagnosed with ST | WFT titers for OXK antigen positive with 1:360 dilution  Malaria smear negative, Leptospirosis, dengue and HIV serology negative  CXR, USG abdomen, ECHO, ECG were normal, Collagen workup was normal, BC and UC sterile | Blood counts normal, LFT deranged, RFT was normal, Urine examination showed hematuria, pyuria | Response within 48 hours to Doxycycline | Atypical manifestation of ST and delayed diagnosis of ST due to poor index of suspicion |
| 4 | Thangaratham et al,2006 | A case of AFI caused by dual and simultaneous infection with Dengue and *Vivax* malaria | 22 year-old-male brought with high grade fever, chills, rigors, cough and 3-day history of high colored urine | Leptospira IgM negative  Widal negative  Anti HAV negative  Anti HEV negative  Dengue IgM positive  Malaria smear-*Vivax* trophozoites noted | Hb=12.6 g% TLC=6100, DLC=P:60 L:38 E:2  ESR=30, PC=100000/microL  SGOT=37.1 IU/L, SGPT=32.4 IU/L  S Bilirubin=1.4mg/dl  S ALP=155U/L  TP=6.8 mg/dl  S albumin=3.4 mg/dl  Creatinine=0.74mg/dl | Treatment with antimalarials | Due to atypical manifestations initial provisional diagnosis of leptospirosis made |
| 5 | Bhat et al,2015 | AFI caused by a mixed infection with Dengue, mixed malaria and Hep A and Hep E | 22-year-old male with fever with yellowish discoloration of the eyes, skin and urine, loose stools | Dengue IgM ELISA positive, Hep E and Hep A antibody testing positive, HIV, Leptospira serology and rickettsial infection negative, anti HCV negative and BC shows no growth, malaria smears mixed infection | PC=12000/microL  Deranged LFT  TB=3076.32micromoles/L  Direct Bilirubin=2192.32micromoles/L  Mildly elevated enzymes  Deranged RFT, creatinine=3.1mg%  Urea=125  Na=129 meq/l, K=4.7 meq/l  CXR=No abnormalities detected  USG=Hepatosplenome-galy | - | Atypical presentatio-n of AFI due to multiple coexisting infections |
| 6 | Jagdishku-mar et al,2016 | AFI caused by mixed infection with Dengue and Typhoid | 3-year-old child with high fever for 5 days and vomiting for 1 day | Dengue IgM and IgG ELISA, both positive  Widal 1:160 titer, BC positive for S. typhi  Malarial smear negative, WFT negative  Urine culture showing no growth | Hb=10.7g/dl  TLC=6500/mm3  Hct=32  ESR=70mm/h  PC=73000/mm3  SGOT=78U/L  SGPT=71U/L | Ceftriaxon-e with supportive therapy | Atypical presentatio-n with Dengue and Typhoid |
| 7 | Kakarapat-hi et al,2014 | *P. vivax* infection mimicking *P. falciparum* malaria as a cause of AFI complicate-d with neurologic-al, hematologi-cal and renal manifestations | 73 year-old-female with complaints of weakness, fever with chills and rigors for 1 day | Malarial smear noted Vivax shizonts and ring forms.  Card test negative for falciparum | Hb=11g/dl, TLC=2600/mm^3^, PC=32000/mm^3^  RFT, Urea=120mg/dl  Creatinine=2.1mg/dl  K=2.7meq/l  LFT normal | Pt discharged after 14 days on primaquine | The importance of differentiat-ng the cause of malaria as treatment is different for the 2 diseases |
| 8 | Madi et al,2014 | Dengue encephaliti-s | 49-year-old male with 6-day history of fever and headache | Malaria test negative  Dengue NS1 antigen positive  Dengue IgM positive  CSF cell count=80cell/ml and all lymphocytes  CSF protein=151.8mg/dl  CSF PCR negative for herpes simplex 1 and 2 | Hb=14.2g/dl, TLC=2800cell/mm^3^, PC=1.24lakh/mm^3^, AST=135U/L, ALT=110U/L  ALP=45U/L  RFT normal | Antiepileptics and supportive treatment | Fever with associated neurological syndromes are the differential diagnosis |
| 9 | Sitalakshm-i et al,2005 | *P. malariae* AFI | 27-year-old male with AFI with history of malaria 6 months ago while in Africa | PBS showing ring forms, band forms and gametocytes of *P.malariae* | Hb=14.6g%, WBC=4300cells/mm^3^, DLC=N:60, L:21, E:1, Myelocytes:2, Band forms:3  PC=23*10^9^/L, PBS showed normocytic, normochromic anemia | Patient recovered with antimalari-al therapy. |  |

**Table 63. Case series: Results of investigations for AFI**

| **Name of study** | **Specific test results** | **Nonspecific tests** | | | |
| --- | --- | --- | --- | --- | --- |
|  |  | **Case 1** | **Case 2** | **Case 3** | **Case 4** |
| Kumar et al,2008 | Case1: Malarial parasite fluorescent test  Case2,3,4: PBS for malaria  Case1,2: diagnosed as vivax malaria  Case3: diagnosed as vivax and falciparum  Case4: vivax and falciparum malaria | CBC, RFT: normal  Urine analysis: normal  TB=17mg/dl  Indirect bilirubin=1.2mg/dl  USG abdomen enlarged spleen: 17cm length with hypoechoic areas, CT confirms splenic infarcts | Hb=10.3g/dl dropped to 9.3g/dl  HCT and coagulation profile: normal  S amylase=normal  RFT: normal  LFT: unconjugated hyperbilirubinemia  TB: 2mg/dl  DB: 0.7mg/dl  CT scan abdomen showed a bulky spleen with perisplenic collection and fluid in bowel loops and pelvis suggestive of hemoperitoneum secondary to splenic rupture | Hb=8.4g/dl, PC=94000/mm^3^, TB=5mg%, DB=4mg%, RFT, creatinine=1.8 mg%, BUN=82mg/dl  MRI abdomen suggestive of splenomegaly with subcapsular infarcts, BC=no growth | Hb=9.2g/dl, PC=95000/mm^3^, Indirect Bilirubin=3.5g/dl, USG Abdomen suggestive of ruptured spleen with hemoperitoneum |
| Katoch et al,2016 | Case1: WFT, OX-19 positive, dengue and malaria testing negative, widal negative, BC showed no growth  Case2: WFT, OX-2 positive, BC showed no growth, widal negative  Case3: WFT, OXK positive, BC showed no growth, dengue and malaria tests negative  Case4: WFT, OX-19, OX-2 positive, BC showed no growth, Dengue and malaria tests negative, widal negative | Normocytic normochromic anemia on PBS, neutrophilic leukocytosis, ESR raised, LFT, RFT were normal, urine examination was normal | Normocytic, normochromic anemia on PBS, leukocytosis, thrombocytopenia, coagulation profile, APTT raised, LFT, RFT were normal, Urine examination normal | Dimorphic anemia on PBS, neutrophilic leukocytosis, thrombocytopenia, ESR raised, LFT, RFT were normal, urine examination was normal | Normocytic, normochromic anemia, leukocytosis, thrombocytosis, ESR raised, CRP raised, raised neutrophil count, LFT, RFT was normal, Urine examination normal |
| Saifudhe-en et al,2012 | Case-1: WFT, OXK:1:5120  HIV, Dengue, leptospirosis, dengue, malaria, hepatitis A, B, E, widal,were negative, CSF opening pressure=260mmH_2_0 with mild lymphocytic pleocytosis(15cells),high protein=60mg and normal glucose  Case-2: WFT, OXK:1:320, CSF pressure=270mm H_2_O, lymphocytic pleocytosis=30 cells, CSF protein=70mg, all other specific tests mentioned above are negative | TLC=8700cells/mm^3,^ DLC=raised neutrophils, normal Hb levels, PC=78000/mm^3^ , ESR=76mm/h, BUL=84mg/dl, creatinine=1.4mg/dl, TB=0.8mg/dl and DB=0.4mg/dl, Albumin=2.8g/dl, TP=6.8g%, SGOT, SGPT=160,185U/L, ALP=82, Urine examination normal, USG abdomen suggestive of hepatosplenomegaly, CXR suggestive of ARDS.  The patient succumbed due to a delayed diagnosis | WBC=9500cells/mm^3^, Thrombocytopenia, serum albumin=3g%, SGOT, SGPT=176,122, RFT, USG abdomen and CXR were normal  Patient was afebrile after 48 hours of Doxycycline therapy |  |  |
| Prasann-an et al,2017 | WFT positive in all cases with titers >1:320 taken as positive  Case1 WFT positive on day 6, Case2 WFT positive on D6, Case3 WFT positive on D9, Case4 WFT positive for WFT on D7 | HB=9.9g%, TLC=12000cells/mm^3^, DLC:P:41, L:56, PC=60000cells/mm^3^, CRP negative, Urea=21, creatinine=0.9mg/dl, Na=134 meq/l, SGOT=27U/l, SGPT=12U/l, ALP=72U/l, Albumin=3.6g% | Hb=10.2g%, TLC=14000 cells/ mm^3^, DLC:P:67, L:32, PC=120000cells/mm^3^, CRP negative, Urea=35, creatinine=0.6mg%, Na=132meq/l, SGOT=34U/l, SGPT=12U/l, ALP=234U/l, Albumin=3.4g% | Hb=8.2g%, TLC=22000 cells/mm^3^, DLC:P:60, L:34, PC=400000cells/mm^3^, CRP positive, Urea=20, creatinine=0.5mg/dl, Na=135meq/l, SGOT=14U/l, SGPT=16U/l, ALP=139U/l, Albumin=2.1g% | Hb=11g%, TLC=8700cells/mm^3^, DLC:P:45, L:52, PC=340000cells/mm^3^, CRP negative, Urea=42, creatinine=0.8mg/dl, Na=129meq/l, SGOT=38U/l, SGPT=24U/l, ALP=169U/l, Albumin=3.8g% |

# **References**

1. Abhilash K, Jeevan J, Mitra S, Paul N, Murugan T, Rangaraj A, et al. Acute undifferentiated febrile illness in patients presenting to a Tertiary Care Hospital in South India: Clinical spectrum and outcome. Journal of Global Infectious Diseases. 2016;8(4):147-54.

2. Chrispal A, Boorugu H, Gopinath KG, Chandy S, Prakash JAJ, Thomas EM, et al. Acute undifferentiated febrile illness in adult hospitalized patients: The disease spectrum and diagnostic predictors - An experience from a tertiary care hospital in South India. Tropical Doctor. 2010;40(4):230-4.

3. Chrispal A, Boorugu H, Gopinath KG, Prakash JAJ, Chandy S, Abraham OC, et al. Scrub typhus: an unrecognized threat in South India – clinical profile and predictors of mortality. Tropical Doctor. 2010;40(3):129-33.

4. Manjunath VG, Hedda S, Vijay Kumar GS, Kumar JK, Murthy DS. Clinical features, laboratory findings and complications of scrub typhus in South Indian children. Journal of Nepal Paediatric Society. 2017;37(1):21-4.

5. Muthaiah B, Thippeswamy T, Kondareddy S, Chikkegowda P. Study of aetiology and outcome in acute febrile illness patients with multiple organ dysfunction syndrome. Journal of Clinical and Diagnostic Research. 2016;10(8):OC16-OC8.

6. Sahana KS, Sujatha R. Clinical profile of dengue among children according to revised WHO classification: analysis of a 2012 outbreak from Southern India. Indian J Pediatr. 2015;82(2):109-13.

7. Viswanathan S, Muthu V, Iqbal N, Remalayam B, George T. Scrub typhus meningitis in South India--a retrospective study. PLoS One. 2013;8(6):e66595.

8. Varghese GM, Trowbridge P, Janardhanan J, Thomas K, Peter JV, Mathews P, et al. Clinical profile and improving mortality trend of scrub typhus in South India. International Journal of Infectious Diseases. 2014;23:39-43.

9. Mathew T, Satishchandra P, Mahadevan A, Nagarathna S, Yasha TC, Chandramukhi A, et al. Neuroleptospirosis - revisited: experience from a tertiary care neurological centre from south India. Indian J Med Res. 2006;124(2):155-62.

10. Muddaiah M, Prakash PS. A study of clinical profile of malaria in a tertiary referral centre in South Canara. Journal of Vector Borne Diseases. 2006;43(1):29-33.

11. Narayanan M, Aravind MA, Thilothammal N, Prema R, Sargunam CS, Ramamurty N. Dengue fever epidemic in Chennai--a study of clinical profile and outcome. Indian pediatrics. 2002;39(11):1027-33.

12. Narayanasamy DK, Arunagirinathan AK, Kumar RK, Raghavendran VD. Clinico - Laboratory Profile of Scrub Typhus - An Emerging Rickettsiosis in India. Indian Journal of Pediatrics. 2016;83(12-13):1392-7.

13. Palanivel S, Nedunchelian K, Poovazhagi V, Raghunadan R, Ramachandran P. Clinical profile of scrub typhus in children. Indian J Pediatr. 2012;79(11):1459-62.

14. Ramabhatta S, Palaniappan S, Hanumantharayappa N, Begum SV. The Clinical and Serological Profile of Pediatric Dengue. Indian Journal of Pediatrics. 2017;84(12):897-901.

15. Stephen S, Sangeetha B, Ambroise S, Sarangapani K, Gunasekaran D, Hanifah M, et al. Outbreak of scrub typhus in Puducherry & Tamil Nadu during cooler months. The Indian Journal of Medical Research. 2015;142(5):591-7.

16. Varghese GM, Abraham OC, Mathai D, Thomas K, Aaron R, Kavitha ML, et al. Scrub typhus among hospitalised patients with febrile illness in South India: Magnitude and clinical predictors. Journal of Infection. 2006;52(1):56-60.

17. Varghese GM, Janardhanan J, Trowbridge P, Peter JV, Prakash JA, Sathyendra S, et al. Scrub typhus in South India: clinical and laboratory manifestations, genetic variability, and outcome. International Journal of Infectious Diseases. 2013;17(11):e981-7.

18. Chacko B, Subramanian G. Clinical, laboratory and radiological parameters in children with dengue fever and predictive factors for dengue shock syndrome. J Trop Pediatr. 2008;54(2):137-40.

19. Poovathingal MA, Nagiri SK, Nagaraja. The emerging trends of falciparum malaria: a study from a tertiary centre in an endemic area of India. Asian Pac J Trop Biomed. 2014;4(Suppl 1):S81-6.

20. Premraj SS, Mayilananthi K, Krishnan D, Padmanabhan K, Rajasekaran D. Clinical profile and risk factors associated with severe scrub typhus infection among non-ICU patients in semi-urban south India. J Vector Borne Dis. 2018;55(1):47-51.

21. Subbalaxmi MV, Madisetty MK, Prasad AK, Teja VD, Swaroopa K, Chandra N, et al. Outbreak of scrub typhus in Andhra Pradesh--experience at a tertiary care hospital. The Journal of the Association of Physicians of India. 2014;62(6):490-6.

22. Vivekanandan M, Mani A, Priya YS, Singh AP, Jayakumar S, Purty S. Outbreak of scrub typhus in Pondicherry. The Journal of the Association of Physicians of India. 2010;58:24-8.

23. Kumar M, Krishnamurthy S, Delhikumar CG, Narayanan P, Biswal N, Srinivasan S. Scrub typhus in children at a tertiary hospital in southern India: clinical profile and complications. Journal of infection and public health. 2012;5(1):82-8.

24. Mathai E, Rolain JM, Verghese GM, Abraham OC, Mathai D, Mathai M, et al. Outbreak of scrub typhus in southern India during the cooler months. Annals of the New York Academy of Sciences. 2003;990:359-64.

25. Palanivel H, Nair S, Subramaniyan A, Ratnam PV, Kanungo R. Dengue virus infection: Need for appropriate laboratory tests for diagnosis and management of the condition in children during an outbreak. Indian Journal of Pathology and Microbiology. 2015;58(3):328-31.

26. Razak A, Sathyanarayanan V, Prabhu M, Sangar M, Balasubramanian R. Scrub typhus in Southern India: are we doing enough? Trop Doct. 2010;40(3):149-51.

27. Moola S, Munn Z, Tufanaru C, Aromataris E, Sears K, Sfetcu R, et al. Systematic reviews of etiology and risk . In: Aromataris E, Munn Z, editors. Joanna Briggs Institute Reviewer's Manual The Joanna Briggs Institute

2017.

1. Serum sodium levels expressed as milliequivalents/litre [↑](#footnote-ref-1)
2. Serum bicarbonate levels expressed in millimoles/litre [↑](#footnote-ref-2)
3. Hematocrit [↑](#footnote-ref-3)
4. International normalized ratio [↑](#footnote-ref-4)
5. Acute respiratory distress syndrome [↑](#footnote-ref-5)
6. Differential leucocyte count [↑](#footnote-ref-6)
7. Renal failure [↑](#footnote-ref-7)
8. Central venous pressure [↑](#footnote-ref-8)
9. Prothrombin time [↑](#footnote-ref-9)
10. Activated partial thromboplastin time [↑](#footnote-ref-10)
